# Further Evidence for the Dark-Ego-Vehicle Principle: Higher Pathological Narcissistic Grandiosity and Virtue Signaling Are Related to Greater Involvement in LGBQ and Gender Identity Activism

**DOI:** 10.1007/s10508-024-03019-9

**Published:** 2024-12-02

**Authors:** Ann Krispenz, Alex Bertrams

**Affiliations:** Division of Educational Psychology, Institute of Educational Science, University of Bern, Fabrikstrasse 8, 3012 Bern, Switzerland

**Keywords:** Activism, Gender identity, LGBQ, Narcissism, Psychopathy

## Abstract

The dark-ego-vehicle principle (DEVP) suggests that individuals with so-called dark personalities (e.g., high narcissistic traits) are attracted to political and social activism that they can repurpose to satisfy their specific ego-focused needs (e.g., signaling moral superiority and manipulating others) instead of achieving prosocial goals. Currently, research on the DEVP is still rare. With two pre-registered studies, we sought further evidence for the DEVP by examining the associations of pathological narcissistic grandiosity with involvement in LGBQ activism (Study 1) and gender identity activism (Study 2). Socioeconomically diverse samples from the USA (Study 1; *N* = 446) and the UK (Study 2; *N* = 837) were recruited online via the research-oriented crowdsourcing platform Prolific. Individuals completed the Pathological Narcissism Inventory as well as measures of involvement in activism. Moreover, we assessed different covariates (e.g., altruism), and potential correlates within the narcissism–activism relationship (i.e., virtue signaling, dominance, and aggression). In addition, we examined potential relationships between other dark personality variables (e.g., psychopathy) and activism. In both samples, higher pathological narcissistic grandiosity was related to greater involvement in activism. As expected, virtue signaling was consistently involved in the relationship between pathological narcissistic grandiosity and activism. However, neither dominance nor aggression was related to individuals’ involvement in activism. The results did also not consistently support a relationship between higher psychopathy and greater involvement in activism. Overall, the findings help to further specify the DEVP.

**Supplementary Information:**

The online version contains supplementary material available at 10.1007/s10508-024-03019-9.

## Introduction

To date, lesbian, gay, bisexual, and queer (LGBQ) people still face severe discrimination, abuse, and violence. Anti-LGBQ behaviors range from derogatory indignities and negative sleights to violent behaviors. In some countries, homophobia and transphobia are even still encouraged by law (e.g., Russian “gay propaganda” law forbids the public portrayal of “non-traditional sexual relations”; Reid, 2023). But even in countries protecting LGBQ rights, LGBQ people are still in danger to be attacked by individuals who consider a homosexual orientation and/or gender diversity as a stark deviation from their values. For example, the Tbilisi gay pride was canceled in 2021 following violent attacks of anti-LGBQ protestors (BBC News, 2021), and in the US Transgender Survey (James et al., 2016), nearly half of the respondents reported to have been verbally attacked (46%) and sexually assaulted at some point in their life (47%). In some cases, LGBQ people are even killed. For example, Koko Da Doll, a 35-year old Black transgender woman, was shot to death in Atlanta (Todd, 2023). Because of such attacks, many individuals engage in LGBQ and gender identity activism to oppose prejudice, discrimination, and violence of the LGBQ minority (Szymanski et al., 2021). In search of the factors driving anti-LGBQ sentiment, previous research mainly focused on individuals attacking the LGBQ community (e.g., Bjork-James, 2019; Bratton et al., 2020; Russell, 2019).

However, the present research is motivated by recent incidents in the context of LGBQ activism which indicate that some activists may engage in the movement not for prosocial but different reasons: For example, in 2021, the so-called anti-TERF^1^ Sussex group campaigned for the termination of Kathleen Stock—a lesbian professor from Sussex University—because of her controversial views on gender self-identification (Woolcock, 2021). In a derogatory way, Stock was called a transphobe, anti-feminist, and anti-queer from “the wrong side of history.” Consequently, the University of Sussex condemned the campaign, but faced a storm of criticism on social media (Kelleher, 2021). Such incidents raise the research question if some of the individuals participating in such conflicts are actually “hijacking” the LGBQ movement for the satisfaction of their own self-centered needs, for example, to signal their moral superiority to other LGBQ activists and to dominate others who are perceived as disloyal or enemies to the cause. With two pre-registered studies, the present research investigates this question based on the recently proposed dark-ego-vehicle principle (DEVP).

### The Dark-Ego-Vehicle Principle

The DEVP states that individuals with pronounced dark personalities may repurpose political and prosocial activism as a vehicle to satisfy their ego-focused needs rather than for the pursuit of prosocial goals and social justice since participation in such activism may give those individuals not only opportunities to gain social status and to engage in social conflicts, but also to display moral superiority and dominate others (Krispenz & Bertrams, 2024b). Whenever using the term dark personalities in this paper, we refer to a set of socially offensive personality traits including narcissism, Machiavellianism, psychopathy, and sadism (i.e., the Dark Tetrad; Paulhus et al., 2021). The present research specifically focused on one of these dark personality traits—narcissism. The contemporary research literature (e.g., Crowe & Miller, 2023; Krizan & Herlache, 2018; Pincus, 2023) conceptualizes narcissism as an interplay of narcissistic vulnerability and grandiosity. While narcissistic vulnerability is characterized by feelings of inadequacy, low self-esteem, and emotional vulnerability, narcissistic grandiosity is defined by “entitled attitudes, an inflated self-image without requisite accomplishments and skills, as well as engagement in regulatory fantasies of unlimited power, superiority, perfection, and adulation,” and “interpersonally exploitative acts, lack of empathy, intense envy, aggression, and exhibitionism” (Wright et al., 2010, p. 468). In the context of the DEVP, we propose that individuals with high levels of narcissistic grandiosity (rather than vulnerability) should be drawn to prosocial activism not for prosocial reasons but for reasons of self-interest. This prediction is based on the following rationale: Firstly, studies show that narcissists actively seek social status (Mahadevan & Jordan, 2022), leadership positions (Grijalva et al., 2015; Nevicka, 2018), and fame (Greenwood et al., 2013)—needs which can be readily satisfied by participation in prosocial activism. Secondly, narcissism has been empirically related to lower empathy (Bushman & Thomaes, 2011) and exploitativeness (i.e., a manipulative interpersonal orientation) (Hepper et al., 2014; Pincus et al., 2009). Concerningly, narcissists seem also to be able to recognize emotions (e.g., anxiety) in others, which may support narcissistic individuals in successfully manipulating others by exploiting their vulnerabilities (Konrath et al., 2014). Further, studies found associations between narcissism and the pretense of altruism and prosocial motivation (Greenwood et al., 2013; Konrath et al., 2016)—characteristics narcissistic individuals may find useful when supposedly participating in prosocial activism while satisfying their own ego-centric needs.

Going beyond the usual focus on nonclinical narcissism in the dark personalities research (Furnham et al., 2013), we further propose that especially the characteristics of pathological narcissistic grandiosity (e.g., Pincus, 2023) may lead to problematic expressions of prosocial activism for the following reasons: Pathological narcissism seems to be more dysfunctional than subclinical narcissism—at least from the narcissists’ perspective. For example, Pincus et al. (2009) found higher pathological narcissism to be associated with lower self-esteem and higher suicidality while so-called normal narcissism was substantially related to higher self-esteem. Further, higher pathological narcissistic grandiosity has been associated with higher hostility, suspiciousness, cognitive/perceptual dysregulation, emotional lability, irresponsibility, eccentricity, and rigid perfectionism when contrasted with more adaptive conceptions of grandiose narcissism (Miller et al., 2014).

It is necessary to stress, however, that the DEVP does not mean that activism is narcissistic per se as many people participate due to altruistic motives (Bertrams & Krispenz, 2024; Krispenz & Bertrams, 2024a). Most importantly, the DEVP does not state that individuals belonging to the respective minority (e.g., members of the LGBQ community) are particularly narcissistic. Rather, the DEVP assumes that some narcissistic individuals may hijack an activist movement, thereby causing serious harm to the movement and the respective minority groups. For example, narcissists may strive for an influential position in the activist movement (cf. Greenwood et al., 2013; Grijalva et al., 2015; Mahadevan & Jordan, 2022) and get access to its financial resources they then use to satisfy their self-centered needs (cf. Campbell et al., 2005). Such self-serving behaviors do not only prevent the activist movement from spending these resources for the prosocial cause but may also cause irreparable financial and reputational harm inevitably reducing public support. Therefore, minority groups and their true allies need to be made aware of the narcissistic “enemies from within” so they can take adequate measures to protect the movement.

### The Present Research

Currently, the DEVP is not yet an elaborated theory. Rather, we understand the present research as part of the process of theory construction methodology (Borsboom et al., 2021)*.* This methodology has a bottom-up nature in which inquiry moves from phenomena to explanation. Currently, there are few empirical studies supporting the DEVP in the context of anti-sexual assault activism (Bertrams & Krispenz, 2024), climate activism (Zacher, 2024), feminist activism (Krispenz & Bertrams, 2024a), and political activism (Fazekas & Hatemi, 2021; Rogoza et al., 2022; see also Krispenz & Bertrams, 2024b). Within Borsboom et al.’s (2021) theoretical circle, the research on the DEVP is nevertheless still at the point where we need to reliably identify the new empirical phenomenon by means of data collection. In the present research, we wanted to add to the existing literature by investigating the validity of the DEVP in yet another context of prosocial activism. For Study 1, we chose the wider context of LGBQ activism and relied on an established measure which allows for the assessment of the involvement in activism aimed at the protection of minority groups including lesbian, gay, bisexual, and queer individuals (Szymanski et al., 2021). Notably, this measure does not differentiate between activism regarding the discrimination of individuals due to their sexual orientation (LGB) versus their gender identity (“queer”). Given the controversy around the topic, we believed it to be critical to additionally focus specifically on gender identity activism. As a consequence, we narrowed our research focus in Study 2 by investigating the DEVP in the context of activism which only aims at “queer” gender identity groups (i.e., transgender and nonbinary people; cf. Atteberry Ash, 2020).

Noteworthy, LGBQ activism is not limited to LGB, transgender, or nonbinary people but also involves so-called allies (Jones et al., 2014). From the perspective of the DEVP, we had no reasons to assume that the narcissism–activism relationship would depend on individuals’ identification as a member of the LGBQ community. Rather, we assumed that some individuals do not participate in LGBQ or gender identity activism to support these minorities but to satisfy ego-focused needs. Accordingly, narcissists can be assumed to participate in any form activism if it serves them as a vehicle to reach their own goals—no matter if they belong to the supported minority group or not.

## Study 1

Study 1 had three distinct goals. As our main research question, we investigated the validity of the DEVP with regard to the relationship between pathological grandiose narcissism and involvement in LGBQ activism. On the basis of the DEVP, we expected higher pathological narcissistic grandiosity to be related to greater involvement in LGBQ activism (pre-registered Hypothesis 1). Furthermore, we predicted that this relationship would hold above and beyond the influence of certain covariates which share some variance with pathological narcissistic grandiosity and/or involvement in LGBQ activism. Firstly, we included pathological narcissistic vulnerability in the analysis since it is substantially correlated with pathological narcissistic grandiosity (Pincus, 2023; Wright et al., 2010). Secondly, we added altruism as a covariate because it mirrors prosocial tendencies to support others at the price of personal cost (Dargan & Schermer, 2022). As LGBQ activism pursues prosocial goals (e.g., equality and social justice; Dunn & Szymanski, 2018; Szymanski et al., 2021), it should naturally attract altruists with the honest desire to protect the LGBQ minority. This notion is supported by research finding altruism to predict involvement in other forms of activism (e.g., Bertrams & Krispenz, 2024; Krispenz & Bertrams, 2024a; see also Strauss Swanson & Szymanski, 2021). Finally, we included age and gender into the analysis. This was done because younger individuals tend to be both—more narcissistic and more engaged in social justice activism than older individuals (Bertrams & Krispenz, 2024)—possibly as a consequence of their more intensive social media use (Hruska & Maresova, 2020). Further, our previous research showed that females are more involved in activism than males (Bertrams & Krispenz, 2024).

As a second research question, we aimed at extending the DEVP research by examining relevant aspects of the nomological network of the narcissism–activism relationship: virtue signaling and social dominance. Virtue signaling entails “symbolic demonstrations that can lead observers to make favorable inferences about the signaler’s moral character” (Ok et al., 2021, p. 1635). LGBQ activism has moral-based elements (e.g., equality and social justice; Dunn & Szymanski, 2018) which may be very attractive to pathological grandiose narcissists as they allow them to signal their self-accredited moral superiority to their ingroup members as well as the public. This argument is supported by research, showing that virtue signaling gets more pronounced with increased narcissism scores (Konrath et al., 2016; Ok et al., 2021). Hence, we predicted higher pathological narcissistic grandiosity to be indirectly related to higher involvement in LGBQ activism via higher virtue signaling (pre-registered Hypothesis 2).

According to Cheng et al. (2010, p. 334), dominance is defined by “the use of intimidation and coercion to attain social status.” We argue that activism may give pathological grandiose narcissists the opportunity to strive for their own ego-focused needs (e.g., the attainment of a leadership position in the movement) by dominating others. Particularly, pathological grandiose narcissists may use aggression and coercion toward individuals they disagree with (i.e., outgroup members or ostensible “ingroup” members who are perceived as disloyal). In line with this argument, previous research showed a significant relationship between narcissism and dominance (Pincus et al., 2009; Zeigler-Hill et al., 2021) and demonstrated that LGBQ activism incorporates methods of aggressive oppression (Wagner & Hayes, 2022). Thus, we predicted higher pathological narcissistic grandiosity to be indirectly related to higher involvement in LGBQ activism via higher dominance (pre-registered Hypothesis 3).

To inform future research on the DEVP, we lastly investigated the relationship between the non-pathological personality variables of the Dark Tetrad (i.e., Machiavellianism, narcissism, psychopathy, and sadism; Paulhus et al., 2021) and involvement in LGBQ activism. As this was done for exploratory reasons, we did not make any specific pre-registered predictions.

## Method

Study 1 was originally designed as a cross-sectional correlational study. With the cross-sectional part of the data collection already completed, we decided to conduct a pre-registered follow-up assessment of involvement of LGBQ activism applying the same measure (Szymanski et al., 2021) 14–15 weeks after the original data collection. This approach was chosen to address the potential problem of common method variance which may arise when self-report data is collected at the same time from the same participants. Such study designs may create variance attributable to the measurement method rather than the constructs the measures represent. In particular, this potentially creates a correlation among the variables generated by their common source, thus causing systematic measurement errors that either inflate or deflate the observed relationships between constructs. As this problem is most prevalent in cross-sectional studies, it can be methodically addressed by longitudinal designs (Chang et al., 2010; Lindell & Whitney, 2001; Wood et al., 2022). Notably, in this way, we did not deviate from our original pre-registered plan, but rather backed it up with supplementary data and analyses.

### Participants

In this article, we only report for each study the power analysis for the statistical test that yielded the highest minimum sample size. As the pre-registered main analysis, Study 1 involved using linear multiple regression to predict involvement in LGBQ activism with five variables (pathological narcissistic grandiosity, pathological narcissistic vulnerability, altruism, age, and gender). Based on typical univariate effects in individual differences research (*r̄* = .19; Gignac & Szodorai, 2016) and social psychology (*r̄* = .21; Richard et al., 2003), a priori sample size targets were calculated using G*Power 3.1 (Faul et al., 2009) assuming approximately 4% variance explained (squared multiple correlation, ρ^2^ = .04), with two-tailed α = .05 and 80% power. The results revealed that a minimum sample of *N* = 371 was required, but we oversampled to compensate for pre-registered data exclusions aiming at a sample of approximately *N* = 500 participants. The participants were recruited from the research-oriented crowdsourcing platform Prolific (https://www.prolific.co). We used Prolific’s option for selecting a representative US sample. The survey link was accessed 526 times. As pre-registered and prior to our analyses, we excluded 80 cases due to incomplete data (*n* = 32), the presence of duplicates (*n* = 19), and a failed attention check (*n* = 29). The final sample consisted of *N* = 446 participants. The main sociodemographic data of this sample is shown in Table 1 (for the full information, see supplementary Table S1).

**Table 1:** Sample demographics for Study 1 (US sample) and Study 2 (UK sample)

	Study 1								Study 2			
	Measurement time 1 *N* = 446				Measurement time 2 *N* = 326				*N* = 837			
	*n*	*M*	*SD*	%	*n*	*M*	*SD*	%	*n*	*M*	*SD*	%
Age (years)^a^		46.20	15.98			48.99	15.34			47.46	15.20	
*Gender*												
Female	225			50.4	157			48.2	438			52.3
Male	215			48.2	165			50.6	394			47.1
Transgender	3			0.7	2			0.6	1			0.1
Nonbinary, genderqueer, genderfluid	–			–	–			–	3			0.4
Other	3			0.7	2			0.6	1			0.1
*Sexual orientation*												
Heterosexual, straight	379			85.0	286			87.7	762			91.0
Homosexual, gay/lesbian	20			4.5	15			4.6	26			3.1
Bisexual	35			7.8	20			6.1	43			5.1
Other	12			2.7	5			1.5	6			0.7
*Self-identification as a LGBQ member*												
Yes	60			13.5	38			11.7	–			–
No	173			83.6	283			86.8	–			–
Unsure	13			2.9	5			1.5	–			–

^a^For Study 2, values of two participants are missing (*n* = 835)

For the re-assessment of involvement in LGBQ activism, we re-invited the original sample via Prolific. The second time of measurement, *N* = 338 individuals participated within the pre-registered interval of seven days. According to the pre-registered criteria and prior to analyses, we excluded *n* = 7 cases due to incomplete data. From the remaining sample of *N* = 331, we were able to match data of *N* = 326 individuals via their Prolific ID. This sample size was sensitive to effects of ρ^2^ = .045 (input parameters for the sensitivity power analysis: H_1_ ≥ H_0_, H_0_ ρ^2^ = 0, α = .05 [two-tailed], 1 − β = .80, number of predictors = 5). Moreover, this sample was sufficiently large to expect correlations to be stable (Schönbrodt & Perugini, 2013). The main sociodemographic data of the matched sample is shown in Table 1 (for the full information, see supplementary Table S1).

### Procedure

Study 1 was announced as an “Attitudes and behaviors study.” All instructions and measures were provided online via the survey software Qualtrics. At the first time of measure, we assessed sociodemographic data (see supplementary Table S1) and all variables in the order described in the measures section. However, the order of the measures of pathological narcissism, altruism, and the dark tetrad was randomized. Each of the variables was shown on a single page. After completing all measures, the participants were thanked, debriefed, and compensated with £2.30 (about 2.90 USD). At the second time of measure, which took place 14–15 weeks after the original data collection, we re-assessed involvement in LGBQ activism and age. The participants were again thanked, debriefed, and compensated with £0.80 (about 1.00 USD).

### Measures

#### Gender and LGBQ Variables

Gender was measured with one item (“What is your gender?”). Answer categories were 1 (*male*), 2 (*female*), 3 (*transgender*), and 4 (*other, please specify*). As only a very small number of participants identified as transgender (*n* = 3) or nonbinary (*n* = 3), those participants could not be included in separate gender categories for the data analyses. Sexual orientation was measured with one item (“What is your sexual orientation?”). Possible answers were 1 (*homosexual, gay/lesbian*), 2 (*bisexual*), 3 (*heterosexual, straight*), and 4 (*other, please specify*). Further, self-identification with the LGBQ community was assessed with one item (“Do you identify yourself as part of the LGBQ community?”). Possible answers were 1 (*yes*), 2 (*no*), or 3 (*unsure*).

#### Attention Check

To check adherence to the instructions, participants were presented with the item “I breathe oxygen every day” on a seven-point scale from 1 (*strongly disagree*) to 7 (*strongly agree*). The participants were explicitly instructed to continue without responding to this item (cf. Jones & Paulhus, 2014). Any response to this item was hence considered a case of failed attention and the data of the responding participants were excluded from the analyses.

#### Pathological Narcissism

Narcissism was measured with the Pathological Narcissism Inventory (PNI; Pincus, 2023; Pincus et al., 2009; Wright et al., 2010). The PNI comprises 52 items which are answered on six-point scales from 0 (*not at all like me*) to 5 (*very much like me*). We calculated mean scores for pathological narcissistic grandiosity (18 items; e.g., “I try to show what a good person I am through my sacrifices”) and vulnerability (34 items; e.g., “It irritates me when people don’t notice how good a person I am”) as well as a mean score for all 52 PNI items. For all scores, higher values represent higher narcissism. The scales demonstrated excellent internal consistencies for the original sample of 446 (grandiosity: McDonald’s ω = .91; vulnerability: McDonald’s ω = .95; PNI total score: McDonald’s ω = .96) and the matched sample of 326 (grandiosity: McDonald’s ω = .91; vulnerability: McDonald’s ω = .96; PNI total score: McDonald’s ω = .96).

#### Dark Tetrad

The dark tetrad variables were measured with the Short Dark Tetrad (SD4; Paulhus et al., 2021). The SD4 assesses the four socially aversive traits of Machiavellianism (seven items; e.g., “Avoid direct conflict with others because they may be useful in the future”), narcissism (seven items; e.g., “I know that I am special because people keep telling me so”), psychopathy (seven items; e.g., “I’ve been in more fights than most people of my age and gender”), and sadism (seven items; e.g., “It’s funny when idiots fall flat on their face”). Agreement with those items was assessed with five-point scales from 1 (*strongly disagree*) to 5 (*strongly agree*). We calculated a mean score for each of the four SD4 dimensions with high scores indicating a high level of the respective trait. The items assessing Machiavellianism showed satisfying to good internal consistency for the original (McDonald’s ω = .78) and the matched sample (McDonald’s ω = .80). The same was true for the items assessing narcissism (McDonald’s ω = .82 and ω = .82), psychopathy (McDonald’s ω = .78 and ω = .78), and sadism (McDonald’s ω = .79 and ω = .80).

#### Altruism

Altruism was measured with the Self-Report Altruism Scale (SRAS; Rushton et al., 1981). The scale consists of 20 items (e.g., “I have given money to a stranger who needed it (or asked me for it)”) assessing self-reported past altruistic behaviors. The items are responded to on five-point scales from 1 (*never*) to 5 (*very often*). Higher mean scores represent a higher tendency to engage in altruistic behaviors. The scale showed good internal consistency for the original (McDonald’s ω = .89) and the matched sample (McDonald’s ω = .89).

#### Dominance

Dominance was assessed with the Dominance and Prestige Scale (Cheng et al., 2010). The scale consists of two subscales measuring prestige (nine items; e.g., “Members of my group respect and admire me”) and dominance (eight items; “I am willing to use aggressive tactics to get my way”). For Study 1, we used only the items measuring dominance which are responded to on seven-point scales from 1 (*not at all*) to 7 (*very much*) with higher mean scores representing a higher dominance. The scale showed good internal consistency for the original (McDonald’s ω = .85) and the matched sample (McDonald’s ω = .85).

#### Virtue Signaling

To assess virtue signaling, we used the Moral Identity Scale (MIS; Aquino & Reed, 2002). The MIS measures two subdimensions of moral identity: internalization (i.e., the extent to which certain morality-related traits are central to one’s self-concept) and symbolization (i.e., the tendency to publicly express one’s moral identity). Participants were presented with a list of nine different positive and morality-related traits (i.e., caring, compassionate, fair, friendly, generous, hardworking, helpful, honest, and kind) and asked to visualize how individuals having these traits would think, feel, and act. Participants’ internalization tendency was measured with five items (e.g., “It would make me feel good to be a person who has these characteristics”). These five items were rated on five-point scales from 1 (*strongly disagree*) to 5 (*strongly agree*). After recoding the inverse items, a mean internalization score was calculated with high scores indicating a high level of internalization of moral identity. The internalization subscale showed satisfying to good internal consistency for the original (McDonald’s ω = .80) and the matched sample (McDonald’s ω = .79). Participants’ symbolization tendency was assessed with five items (e.g., “I often wear clothes that identify me as having these characteristics”). Again, ratings of these five items were made using five-point scales from 1 (*strongly disagree*) to 5 (*strongly agree*). A mean symbolization score was calculated with high scores indicating a high level of symbolization of moral identity. The symbolization subscale showed good internal consistency for the original (McDonald’s ω = .88) and the matched sample (McDonald’s ω = .88).

To calculate virtue signaling scores, we followed the recommended procedure of Ok et al. (2021): Using linear regression analysis, we controlled for participants’ internalized moral identity by predicting their symbolization scores by their internalization scores and saving the unstandardized residuals as virtue signaling scores. Those virtue signaling scores represent how strongly someone tends to publicly demonstrate morality independent of their internalized moral values with higher scores indicating higher levels of virtue signaling.

#### Involvement in LGBQ Activism

Involvement in LGBQ activism was assessed with the LGBQ-modified version of the Involvement of Feminist Activism Scale (Szymanski et al., 2021). The scale consists of 17 items (e.g., “I actively participate in LGBQ organizational, political, social, community, and/or academic activities and events”) and seven-point response scales from 1 (*very untrue of me*) to 7 (*very true of me*). The higher the total mean score the higher an individual is considered to be involved in LGBQ activism. The scale showed excellent internal consistency for the original (McDonald’s ω = .94) and the matched sample (McDonald’s ω = .94).

## Results

### Preliminary Analyses

Means, SDs, and intercorrelations of the analyzed variables are presented in Table 2. For the intercorrelations, we also report bias-corrected and accelerated (BCa) bootstrap 95% confidence intervals (CI) based on 1000 bootstrap samples to address the issues of non-normally distributed values and heteroscedasticity (Field, 2018). As we relied on self-report questionnaires, we controlled for common method variance (Chang et al., 2010). For this purpose, we conducted the Harman single-factor test as described in Zhang et al. (2022) and included all items measuring narcissism and involvement in LGBQ activism in an unrotated principal component analysis. This allowed us to test if only one component emerges from this analysis and/or if the first of the found component explains the vast majority of the variation (> 40%)—as each of these cases would indicate the existence of severe common method bias. In the analysis, eleven components emerged explaining 58.6% of the variance. Further, the first component explained only 26.2% of the variation, which is below the critical value. This result suggested that common method bias did not seriously affect the examined relationship.

**Table 2:** Descriptive statistics and zero-order-correlations for Study 1

Variable			Correlations [BCa 95% CIs]														
	*M*	*SD*	1	2	3	4	5	6	7	8	9	10	11	12	13	14	15
1. Involvement in LGBQ activism (original data collection)	1.85	1.07	–														
2. Pathological narcissistic grandiosity	2.12	0.93	.22*** [.14, .31]	–													
3. Pathological narcissistic vulnerability	1.88	0.92	.13** [.04, .22]	.58*** [.50, .64]	–												
4. Pathological narcissism total score	1.93	0.85	.18*** [.10, .26]	.80*** [.77, .84]	.95*** [.93, .96]	–											
5. Altruism	2.59	0.59	.20*** [.10, .29]	.08 [–.01, .17]	− .18*** [–.27, –.10]	− .11* [–.20, –.02]	–										
6. Dominance	2.53	1.08	.08 [–.01, .17]	.54*** [.46, .61]	.42*** [.34, .49]	.49*** [.42, .56]	.04 [–.04, .13]	–									
7. Virtue signaling^a^	0.00	0.93	.20*** [.11, .28]	.27*** [.17, .37]	.06 [–.05, .17]	.14** [.04, .24]	.23*** [.14, .31]	.11* [.01, .20]	–								
8. Dark Tetrad—Machiavellianism	3.15	0.69	− .03 [–.14, .08]	.41*** [.34, .48]	.42*** [.33, .49]	.44*** [.37, .51]	− .12* [–.20, –.03]	.44*** [.37, .51]	.04 [–.07, .14]	–							
9. Dark Tetrad—narcissism	2.58	0.77	.16*** [.07, .25]	.63*** [.57, .70]	.17*** [.08, .25]	.35*** [.27, .43]	.20*** [.11, .28]	.59*** [.53, .64]	.28*** [.19, .37]	.29*** [.20, .37]	–						
10. Dark Tetrad—psychopathy	1.85	0.66	.21*** [.11, .32]	.39*** [.32, .47]	.35*** [.26, .42]	.39*** [.31, .48]	.02 [–.08, .12]	.49*** [.41, .56]	.09 [–.02, .19]	.25*** [.17, .33]	.41*** [.33, .48]	–					
11. Dark Tetrad—sadism	2.24	0.80	− .01 [–.11, .09]	.40*** [.32, .48]	.31*** [.22, .39]	.37*** [.29, .45]	− .10* [–.20, –.01]	.43*** [.35, .51]	− .04 [–.14, .06]	.44*** [.36, .51]	.35*** [.26, .43]	.56*** [.49, .63]	–				
12. Age (in years)	46.20	15.98	− .20*** [–.29, –.11]	− .34*** [–.41, –.26]	− .33*** [–.40, –.25]	− .37*** [–.44, –.30]	.31*** [.22, .39]	− .17*** [–.25, –.10]	− .01 [–.09, .08]	− .26*** [–.34, –.17]	− .12** [–.20, –.05]	− .23*** [–.32, –.15]	− .30*** [–.38, –.22]	–			
13. Gender^b^	–	–	.14** [.04, .23]	− .10* [–.18, –.02]	.03 [–.07, .12]	− .02 [–.11, .06]	.01 [–.08, .09]	− .15** [–.24, –.06]	.13** [.04, .21]	− .17*** [–.26, –.07]	− .12* [–.21, –.03]	− .21*** [–.30, –.12]	− .37*** [–.45, –.29]	.05 [–.04, .14]	–		
14. Sexuality/gender^c^	–	–	.44*** [.34, .53]	.07 [–.03, .17]	.10* [.01, .19]	.10* [.00, .20]	− .06 [–.15, .04]	.07 [–.03, .17]	− .05 [–.14, .04]	.04 [–.05, .14]	.00 [–.10, .09]	.15*** [.06, .26]	.07 [–.02, .16]	− .31*** [–.38, –.22]	.03 [–.06, .13]	–	
15. Self-identification as member of LGBQ community^d^	–	–	.47*** [.37, .58]	.09 [–.01, .20]	.10*–[.01, .20]	.11* [.00, .21]	− .03 [–.13, .08]	.06 [–.03, .16]	− .05 [–.16, .06]	.03 [–.08, .13]	.03 [–.06, .13]	.16*** [.06, .27]	.05 [–.04, .15]	− .28*** [–.36, –.20]	.02 [–.08, .12]	.91*** [.84, .96]	–
16. Involvement in LGBQ activism (follow-up)	1.88	1.07	.82*** [.74, .87]	.21*** [.10, .31]	.13* [.00, .25]	.17** [.05, .29]	.20*** [.09, .31]	.10 [.00, .21]	.28*** [.17, .37]	.01 [–.11, .14]	.18** [.07, .28]	.20*** [.08, .32]	.03 [–.07, .14]	− .21*** [–.30, –.11]	.05 [–.06, .15]	.50*** [.38, .61]	.53*** [.37, .63]

*N* = 446 (original data collection), *N* = 326 (follow-up data collection). Displayed are Pearson product–moment correlation coefficients. BCa 95% CI = bias-corrected and accelerated bootstrap 95% confidence intervals based on 1000 bootstrap samples. The pathological narcissism total score is comprised of the averaged 52 Pathological Narcissism Inventory (Pincus et al., 2009) items. For all correlations with gender, transgender and nonbinary participants could not be included in own gender categories due to their small numbers resulting in *n* = 440 (original data collection) and *n* = 322 (follow-up data collection). For correlations with self-identification as member of the LGBQ community: *n* = 433 (original data collection), as participants who indicated being “unsure” were not included (the *n* of the follow-up data collection remained unaffected in this regard)

^a^Scores were calculated using unstandardized residuals

^b^Coding: 1 = male, 2 = female

^c^Coding: 1 = heterosexual and male/female, 2 = nonheterosexual and/or not male/female (i.e., transgender or nonbinary)

^d^Coding: 1 = no, 2 = yes

^*^p ≤ .05. **p ≤ .01. ***p ≤ .001. The *p* values refer to two-tailed testing

### Pre-Registered Main Analyses

In our main pre-registered hypothesis, we assumed that pathological narcissistic grandiosity would be related to higher involvement in LGBQ activism (pre-registered Hypothesis 1). As expected, a bivariate correlation analysis revealed that higher pathological narcissistic grandiosity was related to more pronounced involvement in LGBQ activism: *r* = .22, *p* < .001. An alternative analysis using Spearman’s nonparametric correlation coefficient revealed a very similar result: ρ = .23, *p* < .001. Similarly, pathological narcissistic grandiosity was correlated with involvement in LGBQ activism measured at the follow-up (*r* = .21, *p* < .001; ρ = .22, *p* < .001). For all results, see Table 2.

Next, we regressed involvement in LGBQ activism on pathological narcissistic grandiosity for both times of measure with multiple regression analyses and included the pre-registered covariates (i.e., pathological narcissistic vulnerability, altruism, age, and gender). Regarding the original sample, the effect size of the relationship between involvement in LGBQ activism and pathological narcissistic grandiosity was statistically significant (β = .13, *p* = .025). In addition, involvement in LGBQ activism was related to altruism (β = .25, *p* < .001), age (β = –.24, *p* < .001), and gender (β = .16, *p* < .001), but not pathological narcissistic vulnerability (β = .01, *p* = .800). A multiple regression analysis predicting involvement in LGBQ activism measured at the follow-up yielded comparable results. For all results of these analyses, see Table 3.^2^

**Table 3:** Multiple regression analysis for involvement in LGBQ activism measured at the original and the follow-up data collection for Study 1

Variable	Original data collection (*n* = 440)						Follow-up data collection (*n* = 322)					
	*B*	BCa 95% CI for *B*	*SE B*	β	*t*	*p*	*B*	BCa 95% CI for *B*	*SE B*	β	*t*	*p*
Constant	0.54	–0.01, 1.12	0.31	–	1.72	.086	0.87	0.14, 1.67	0.38	–	2.31	.022
Pathological narcissistic grandiosity	0.15	0.02, 0.30	0.07	.13	2.26	.025	0.16	0.03, 0.31	0.08	.14	2.05	.042
Pathological narcissistic vulnerability	0.02	− 0.10, 0.13	0.07	.01	0.25	.800	0.02	− 0.13, 0.17	0.08	.02	0.26	.799
Altruism	0.45	0.27, 0.63	0.09	.25	5.11	< .001	0.43	0.23, 0.67	0.10	.25	4.32	< .001
Age (in years)	− 0.02	− 0.02, − 0.01	0.00	− .24	− 4.75	< .001	− 0.02	− 0.02, − 0.01	0.00	− .24	− 4.08	< .001
Gender	0.34	0.14, 0.53	0.09	.16	3.56	< .001	0.19	− 0.03, 0.41	0.11	.09	1.73	.085

The sample sizes are reduced as transgender and nonbinary participants could not be included in own gender categories due to their small numbers. Parameters for the overall model (original data collection): *F*(5, 434) = 15.17, *p* < .001, *R*^2^ = .15, adjusted *R*^2^ = .14. Parameters for the overall model (follow-up data collection): *F*(5, 316) = 9.62, *p* < .001, *R*^2^ = .13, adjusted *R*^2^ = .12. BCa 95% CI = bias-corrected and accelerated bootstrap 95% confidence intervals based on 1000 bootstrap samples. Coding for gender: 1 = male, 2 = female. The *p* values in the table refer to two-tailed testing

### Pre-Registered Secondary Analyses

Next, we examined whether there was an indirect association between pathological narcissistic grandiosity and involvement in LGBQ activism via virtue signaling (pre-registered Hypothesis 2) and/or dominance (pre-registered Hypothesis 3). For this purpose, we employed the analysis software PROCESS (model 4, single mediator model; Hayes, 2022). In the following, we report 95% bootstrapped confidence intervals (BCI) and standard errors based on 1000 bootstrap samples and the percentile method. As predicted, results showed a statistically significant effect of pathological narcissistic grandiosity on virtue signaling (*a* = 0.27, 95% BCI [0.17, 0.37]) as well as a direct (*c*’ = 0.20, 95% BCI [0.10, 0.31]) and indirect effect (*ab* = 0.05, 95% BCI [0.02, 0.09]) of pathological narcissistic grandiosity on involvement in LGBQ activism via virtue signaling. Also, there was a statistically significant effect of pathological narcissistic grandiosity on dominance (*a* = 0.62, 95% BCI [0.53, 0.72]) and involvement in LGBQ activism (*c*’ = 0.28, 95% BCI [0.15, 0.41]). There was, however, no significant indirect path between pathological narcissistic grandiosity and involvement in LGBQ activism via dominance (*ab* = –0.03, 95% BCI [–0.10, 0.04]). Very similar findings were obtained when we replaced the original measure of involvement in LGBQ activism with its follow-up measure. For all results of these analyses, see Table 4.

**Table 4:** Multiple regression analysis for involvement in LGBQ activism via dominance and virtue signaling for Study 1

Variable	Original data collection (*N* = 446)									
	Dominance					Involvement in LGBQ activism				
	*B*	95% BCI for *B*	*SE B*	*t*	*p*	*B*	95% BCI for *B*	*SE B*	*t*	*p*
Constant	1.21	1.01, 1.40	0.11	11.23	< .001	1.38	1.15, 1.63	0.14	9.93	< .001
Pathological narcissistic grandiosity	0.62	0.53, 0.72	0.05	13.43	< .001	0.28	0.15, 0.41	0.06	4.49	< .001
Dominance	–	–	–	–	–	–0.05	–0.16, 0.06	0.05	–0.99	.321
	*R*^2^ = .29 *F*(1, 444) = 180.48, *p* < .001					*R*^2^ = .05 *F*(2, 443) = 11.50, *p* < .001				

	Virtue signaling					Involvement in LGBQ activism				
	*B*	95% BCI for *B*	*SE B*	*t*	*p*	*B*	95% BCI for *B*	*SE B*	*t*	*p*
Constant	–0.58	–0.80, –0.36	0.11	–5.49	< .001	1.42	1.22, 1.65	0.13	11.25	< .001
Pathological narcissistic grandiosity	0.27	0.17, 0.37	0.05	5.99	< .001	0.20	0.10, 0.31	0.05	3.72	< .001
Virtue signaling	–	–	–	–	–	0.17	0.07, 0.27	0.05	3.08	.002
	*R*^2^ = .07 *F*(1, 444) = 35.90, *p* < .001					*R*^2^ = .07 *F*(2, 443) = 15.95, *p* < .001				

	Follow-up data collection (*N* = 326)									
	Dominance					Involvement in LGBQ activism				
	*B*	95% BCI for *B*	*SE B*	*t*	*p*	*B*	95% BCI for *B*	*SE B*	*t*	*p*
Constant	1.26	1.03, 1.49	0.12	10.40	< .001	1.40	1.13, 1.72	0.16	8.59	< .001
Pathological narcissistic grandiosity	0.61	0.51, 0.72	0.05	11.56	< .001	0.25	0.10, 0.40	0.07	3.39	< .001
Dominance	–	–	–	–	–	–0.02	–0.15, 0.11	0.06	–0.25	.802
	*R*^2^ = .29 *F*(1, 324) = 133.57, *p* < .001					*R*^2^ = .04 *F*(2, 323) = 7.53, *p* < .001				

	Virtue signaling					Involvement in LGBQ activism				
	*B*	95% BCI for *B*	*SE B*	*t*	*p*	*B*	95% BCI for *B*	*SE B*	*t*	*p*
Constant	–0.60	–0.87, –0.35	0.12	–4.90	< .001	1.54	1.29, 1.81	0.14	10.80	< .001
Pathological narcissistic grandiosity	0.29	0.17, 0.41	0.05	5.37	< .001	0.16	0.04, 0.28	0.06	2.60	.010
Virtue signaling	–	–	–	–	–	0.27	0.14, 0.39	0.06	4.25	< .001
	*R*^2^ = .08 *F*(1, 324) = 28.88, *p* < .001					*R*^2^ = .10 *F*(2, 323) = 16.95, *p* < .001				

95% BCI = bootstrap 95% confidence intervals based on 1000 bootstrap samples. The *p* values in the table refer to two-tailed testing

For exploratory reasons, we regressed involvement in LGBQ activism simultaneously on each of the four Dark Tetrad variables. Results revealed that higher Dark Tetrad narcissism (β = .13, *p* = .013) and higher Dark Tetrad psychopathy (β = .28, *p* < .001) were related to higher involvement in LGBQ activism over and above the other Dark Tetrad variables. In contrast, Dark Tetrad sadism (β = –.19, *p* = .001) was negatively related to involvement in LGBQ activism while Dark Tetrad Machiavellianism (β = –.05, *p* = .304) was unrelated to involvement in LGBQ activism. When repeating these analyses with involvement in LGBQ activism measured at the follow-up, we found very similar results. For all results of this analysis, see Table 5.

**Table 5:** Multiple regression analysis for involvement in LGBQ activism measured at the original and the follow-up data collection for Study 1

Variable	Original data collection (*N* = 446)						Follow-up data collection (*N* = 326)					
	*B*	BCa 95% CI for *B*	*SE B*	β	*t*	*p*	*B*	BCa 95% CI for *B*	*SE B*	β	*t*	*p*
Constant	1.38	0.91, 1.84	0.25	–	5.43	< .001	1.24	0.68, 1.86	0.29	–	4.23	< .001
Dark Tetrad—Machiavellianism	− 0.08	− 0.26, 0.09	0.08	− .05	− 1.03	.304	− 0.03	− 0.24, 0.18	0.09	− .02	− 0.35	.726
Dark Tetrad—narcissism	0.18	0.05, 0.31	0.07	.13	2.51	.013	0.20	0.04, 0.37	0.09	.15	2.35	.020
Dark Tetrad—psychopathy	0.45	0.26, 0.66	0.09	.28	4.90	< .001	0.39	0.14, 0.61	0.11	.24	3.48	< .001
Dark Tetrad—sadism	− 0.26	− 0.41, − 0.10	0.08	− .19	− 3.22	.001	− 0.22	− 0.42, − 0.04	0.10	− .17	− 2.26	.024

Parameters for the overall model (original data collection): *F*(4, 441) = 10.12, *p* < .001, *R*^2^ = .08, adjusted *R*^2^ = .08. Parameters for the overall model (follow-up data collection): *F*(4, 321) = 5.95, *p* < .001, *R*^2^ = .07, adjusted *R*^2^ = .06. BCa 95% CI = bias-corrected and accelerated bootstrap 95% confidence intervals based on 1000 bootstrap samples. The *p* values in the table refer to two-tailed testing

## Discussion

The results of Study 1 provided first evidence that higher pathological narcissistic grandiosity was related to higher involvement in LGBQ activism for both times of measurement. The narcissism–activism relationship also held above the pre-registered covariates (i.e., pathological narcissistic vulnerability, altruism, age, and gender). As expected, involvement in LGBQ activism was also significantly predicted by individuals’ self-reported altruism. With regard to the nomological network of the narcissism–activism relationship, we found that virtue signaling may be a vital narcissistic motivation involved in the narcissism–activism relationship. Unexpectedly, social dominance did not play a significant role in the narcissism–activism relationship.

The exploration of the relationship between the non-pathological variables of the Dark Tetrad (Paulhus et al., 2021) and LGBQ activism yielded interesting results. Firstly, the results showed that higher Dark Tetrad narcissism was related to higher involvement in LGBQ activism over and above the other Dark Tetrad variables. Secondly, we found greater involvement in LGBQ activism to be related to higher Dark Tetrad psychopathy and lower Dark Tetrad sadism. Thirdly, involvement in LGBQ activism and Dark Tetrad Machiavellianism were not stably interrelated.

The analyses with involvement in LGBQ activism measured 14–15 weeks after the original measurement revealed very similar findings for the analyses reported above. Therefore, it is unlikely that common method variance (Chang et al., 2010) is accountable for the found relationships. In addition, the Harman single-factor test (Zhang et al., 2022) supported the view that common method bias did not occur in Study 1.

## Study 2

Study 2 was conducted to investigate the narcissism–activism relationship in the narrower context of gender identity activism. Based on the DEVP, we expected higher pathological narcissistic grandiosity to be related to greater involvement in gender identity activism (pre-registered Hypothesis 4). Similar to Study 1, we predicted that higher pathological narcissistic grandiosity would be indirectly related to greater involvement in gender identity activism via higher virtue signaling (pre-registered Hypothesis 5).

As a novel aspect, we further investigated the indirect role of aggression in the narcissism–activism relationship. In Study 2, we defined human aggression as a behavior that is intended to harm others who want to avoid the harm (Anderson & Bushman, 2002). We predicted that higher pathological narcissistic grandiosity would also be indirectly related to greater involvement in gender identity activism via higher aggression (pre-registered Hypothesis 6) based on the following rationale: For many individuals, discussions around (their own or others’) gender identity are regarded as a very sensitive and moral-emotional topic with a high potential for social conflict. For instance, prominent conflicts around gender identity—such as the recent fallout the beer brand Bud Light faced after partnering with Dylan Mulvaney—can generate antagonistic groups in the (social) media, which emotionally (if not aggressively) dispute who is in the right (Caruso, 2023, June 30). In line with the DEVP, we assumed that engagement in such emotional conflicts may satisfy narcissistic desires for seeking thrill and acting out reactive anger when someone disagrees with one’s own opinion. This assumption is also supported by a recent meta-analysis (Kjærvik & Bushman, 2021) which found a robust link between narcissism and numerous forms of aggression.

Based on the results of Study 1, we further investigated the relationship between psychopathy (i.e., the dimension of “enjoy hurting”) and involvement in gender identity activism. In the present research, we relied on a triarchic model of psychopathy (Patrick, 2010). In its original version, the model proposes that psychopathy is characterized by (1) boldness, (2) meanness, and (3) disinhibition. Later research (Pink, et al., 2022; Roy et al., 2020) revealed a need to further differentiate these three domains proposing a seven-factor model. According to this model, boldness is characterized by being a (1) leader (i.e., influencing others and taking charge), (2) stress immune (i.e., experiencing a lack of fear and the ability to bounce back), and (3) having a positive self (i.e., feelings of optimism and self-confidence). Meanness is characterized by being (4) callous (i.e., showing a lack of empathy and sensitivity regarding others), and (5) the enjoyment of hurting others (i.e., through aggressive and cruel behaviors). Lastly, disinhibition is characterized by being (6) impulsive (i.e., impatient, needy for instant gratification, and lacking of planning), and (7) antisocial (i.e., conning and irresponsible).

For Study 2, we expected in particular the meanness component “enjoy hurting” to be associated with higher involvement in gender identity activism (pre-registered Hypothesis 7) via higher aggression (pre-registered Hypothesis 8). These predictions were triggered by an incident in which a transgender activist told other participants of a Trans + Pride rally “if you see a TERF, punch them in the [expletive] face” (Reynolds, 2023, 13 August). From the perspective of the DEVP, we propose that such behaviors are indicative of the opportunities that gender identity activism may give psychopathic individuals to inflict harm on others (cf. Neumann et al., 2015)—particularly on those who are perceived as threats to the gender identity movement. This way, psychopathic individuals can not only elevate their own status within the movement, but also send a clear message to those who oppose the movement’s aims by threatening retribution or punishment (doxing, harassment, etc.) for advocating “incorrect” positions. This argument is supported by empirical evidence which specifically linked the psychopathic characteristic “enjoy hurting” to proactive and reactive aggression (Pink et al., 2022; see also Donnellan & Burt, 2016; Gray et al., 2019).

## Method

### Participants

Study 2 involved using linear multiple regression to predict involvement in gender identity activism with nine variables (pathological narcissistic grandiosity, pathological narcissistic vulnerability, and the seven psychopathy facets). Using G*Power 3.1 (Faul et al., 2009), again assuming approximately 4% variance explained (squared multiple correlation, ρ^2^ = .04; cf. Gignac & Szodorai, 2016; Richard et al., 2003), with two-tailed α = .025 and 80% power results of an a priori power analysis revealed a minimum sample of *N* = 510 was required. The significance level of .025 was based on a Bonferroni correction (i.e., the conventional significance level of .05/2) as two relationships were of central interest in this study (i.e., [1] between pathological narcissistic grandiosity and involvement in gender identity activism and [2] between the psychopathy aspect “enjoy hurting” and involvement in gender identity activism). To compensate for pre-registered data exclusions, we oversampled and aimed for a sample of about *N* = 1000 participants. Again, the participants were recruited from Prolific (https://www.prolific.co). This time we used Prolific’s option for selecting a representative UK sample. The survey link was accessed 1083 times. According to the pre-registered criteria and prior to analyses, we excluded 246 cases due to incomplete data (*n* = 81), the presence of duplicates (*n* = 51), and a failed attention check (*n* = 114). The final sample consisted of *N* = 837 participants; their main sociodemographic data are shown in Table 1 (for the full sample information, see supplementary Table S1).

### Procedure

Study 2 was announced as an “Attitudes and behaviors study.” All instructions and measures were provided online via the survey software Qualtrics. In a first step, demographic data was assessed (see supplementary Table S1). Next, the participants completed all measures in the same sequence that they are reported down below. In random sequence, the participants first completed either the measure for pathological narcissism or psychopathy. Each variable was shown on a single page. At the end, the participants were thanked, debriefed, and compensated each with £2.25 (about 2.85 USD).

### Measures

#### Gender and Sexual Orientation

Gender was measured with one item (“What is your gender?”). Answer categories were 1 (*male*), 2 (*female*), 3 (*transgender*), 4 (*nonbinary, genderqueer, genderfluid*), and 5 (*other, please specify*). As there was a very small sample of participants identifying as transgender (*n* = 3) or nonbinary/genderqueer/genderfluid (*n* = 1), those participants could not be included into separate gender categories for the data analyses. Sexual orientation was measured with one item (“What is your sexual orientation?”). Possible answers were 1 (*bisexual*), 2 (*heterosexual, straight*), 3 (*homosexual, gay/lesbian*), and 4 (*other, please specify*).

#### Attention Check

We used the same attention check as in Study 1. Again, any response to the item “I breathe oxygen every day” led to an exclusion from the data analyses adhering to the pre-registered criteria for data exclusions.

#### Pathological Narcissism

Narcissism was again measured with the PNI (Pincus, 2023; Pincus et al., 2009; Wright et al., 2010). All three scales demonstrated good to excellent internal consistencies (grandiosity: McDonald’s ω = .89; vulnerability: McDonald’s ω = .95; PNI total score: McDonald’s ω = .96).

#### Psychopathy

Psychopathy was measured with the Triarchic Psychopathy Measure (TriPM; Patrick, 2010). The TriPM assesses seven subdimensions of psychopathy: leader (five items; e.g., “I’m a born leader”), stress immune (six items; e.g., “I’m afraid of far fewer things than most people”), positive self (three items; e.g., “I’m optimistic more often than not”), callous (five items; e.g., “I don’t have much sympathy for people”), enjoy hurting (eight items; e.g., “I insult people on purpose to get a reaction from them”), impulsive (six items; e.g., “I often act on immediate needs”), and antisocial (seven items; e.g., “I have missed work without bothering to call in”). Agreement with those items was assessed with four-point scales from 1 (*true*) to 4 (*false*). Before calculating a mean score for each of the seven TriPM dimensions, we inversed all items so that high scores indicate a high level of the respective psychopathic dimension. The items assessing leadership (McDonald’s ω = .85) and callousness (McDonald’s ω = 0.88) showed good internal consistency. Satisfying internal consistencies were found for the items assessing immunity to stress (McDonald’s ω = .78), positive self (McDonald’s ω = .75), enjoyment of hurting (McDonald’s ω = .76), impulsivity (McDonald’s ω = .69), and antisociality (McDonald’s ω = .68).

#### Virtue Signaling

Virtue signaling was again assessed with the MIS (Aquino & Reed, 2002) and the calculation procedure suggested by Ok et al. (2021). The internalization subscale (McDonald’s ω = 0.80) as well as the symbolization subscale (McDonald’s ω = .86) showed good internal consistency. Again, we used linear regression analyses to predict participants’ symbolization scores by their respective internalization scores and saving the unstandardized residuals as participants’ virtue signaling scores.

#### Aggression

Aggression was measured with the Brief Aggression Questionnaire (BAQ; Webster et al., 2015). The BAQ comprises 12 items measuring four dimensions: physical aggression (e.g., “Given enough provocation, I may hit another person”), anger (e.g., “Sometimes I fly off the handle for no good reason”), verbal aggression (e.g., “When people annoy me, I may tell them what I think of them”), and hostility (e.g., “When people are especially nice, I wonder what they want”). The items are responded to on seven-point scales from 1 (*extremely uncharacteristic of me*) to 7 (*extremely characteristic of me*). For the data analyses, we calculated a total mean score for all 12 items. Higher mean scores represent a higher level of self-reported aggression. The scale showed good internal consistency (McDonald’s ω = .82).

#### Involvement in Gender Identity Activism

Involvement in gender identity activism was assessed with the adapted version of the Involvement of Feminist Activism Scale (Atteberry Ash, 2020). Instructions read:

For each of the following statements, indicate to what degree it describes your involvement in the stated pro-transgender/pro-nonbinary gender identities activity. For this study, “transgender” includes anyone whose current gender is different than the one assigned at birth. “Non-binary” includes all gender identities that are not solely male or female.

After reading the instructions, participants answered 16 items (e.g., “I am involved in research, writing, and/or speaking about transgender or non-binary people”) on seven-point response scales from 1 (*very untrue of me*) to 7 (*very true of me*). The higher the total mean score the higher an individual can be considered to be involved in gender identity activism. The scale showed excellent internal consistency (McDonald’s ω = .91).

## Results

### Preliminary Analyses

Means, standard deviations, and intercorrelations of the analyzed variables are presented in Table 6. Again, we controlled for common method variance (cf. Chang et al., 2010) using the Harman single-factor test as described in Zhang et al. (2022) including all items measuring narcissism and involvement in gender identity activism in an unrotated principal component analysis. In the analysis, 12 components emerged explaining 56.4% of the variance. Further, the first component explained only 25.5% of the variation, which is below the critical value. The results of this test suggested that common method bias did also not seriously affect the examined relationships in Study 2.

**Table 6:** Descriptive statistics and zero-order-correlations for Study 2

			Correlations [BCa 95% CIs]													
Variable	*M*	*SD*	1	2	3	4	5	6	7	8	9	10	11	12	13	14
1. Involvement in gender identity activism	1.53	0.77	–													
2. Pathological narcissistic grandiosity	2.22	0.83	.19*** [.12, .26]	–												
3. Pathological narcissistic vulnerability	2.08	0.87	.14*** [.08, .20]	.58*** [.53, .63]	–											
4. Pathological narcissism total score	2.12	0.80	.18*** [.12, .24]	.80*** [.77, .82]	.95*** [.94, .96]	–										
5. TriPM leader	2.26	0.70	.08* [.02, .14]	.43*** [.38, .49]	–.05 [–.12, .03]	.11** [.04, .18]	–									
6. TriPM stress immune	2.29	0.63	–.06 [–.13, .02]	.02 [–.05, .09]	–.41*** [–.46, –.36]	–.31*** [–.37, –.26]	.45*** [.39, .50]	–								
7. TriPM positive self	2.80	0.70	–.02 [–.09, .05]	–.03 [–.10, .04]	–.46*** [–.51, –.41]	–.36*** [–.42, –.30]	.47*** [.41, .52]	.50*** [.44, .55]	–							
8. TriPM callous	1.66	0.60	− .16*** [–.22, –.09]	–.10** [–.18, –.02]	.08* [.01, .15]	.00 [–.07, .07]	–.04 [–.11, .02]	.15*** [.07, .22]	–.14*** [–.21, –.07]	–						
9. TriPM enjoy hurting	1.46	0.44	–.03 [–.09, .03]	.33*** [.26, .39]	.21*** [.15, .30]	.26*** [.21, .32]	.26*** [.20, .32]	.28*** [.22, .34]	–.05 [–.12, .02]	.39*** [.32, .45]	–					
10. TriPM impulsive	2.27	0.53	.11** [.04, .18]	.30*** [.24, .35]	.49*** [.43, .54]	.47*** [.41, .53]	–.03 [–.10, .04]	–.26*** [–.32, –.19]	–.41*** [–.47, –.35]	.11** [.03, .18]	.30*** [.23, .37]	–				
11. TriPM antisocial	1.28	0.40	.11** [.04, .18]	.26*** [.21, .32]	.29*** [.23, .36]	.31*** [.25, .37]	.11** [.04, .18]	.01 [–.06, .08]	− .25*** [–.31, –.18]	.19*** [.13, .27]	.46*** [.38, .52]	.39*** [.32, .45]	–			
12. Virtue signaling^a^	0.00	0.88	.20*** [.13, .27]	.25*** [.19, .31]	.12*** [.04, .20]	.18*** [.10, .26]	.16*** [.09, .23]	.07 [–.01, .13]	.10** [.03, .17]	− .09** [–.16, –.03]	.10** [.04, .17]	.08* [.01, .15]	.01 [–.06, .08]	–		
13. Aggression	2.99	1.01	.03 [–.04, .09]	.36*** [.30, .43]	.57*** [.52, .62]	.54*** [.49, .60]	.05 [–.02, .12]	− .11*** [–.19, –.05]	–.41*** [–.47, –.35]	.33*** [.27, .40]	.45*** [.39, .50]	.50*** [.44, .55]	.40*** [.34, .46]	.04 [–.03, .11]	–	
14. Age (in years)^b^	47.46	15.20	–.26*** [–.32, –.20]	–.38*** [–.44, –.33]	–.32*** [–.38, –.27]	–.39*** [–.44, –.33]	–.03 [–.10, .04]	.13*** [.06, .19]	.14*** [.08, .20]	–.05 [–.12, .02]	–.25*** [–.31, –.19]	–.29*** [–.35, –.23]	–.22*** [–.27, –.16]	− .23*** [–.30, –.17]	–.30*** [–.36, –.24]	–
15. Gender^c^	–	–	.09* [.02, .15]	–.14*** [–.21, –.28]	.06 [.00, .12]	.00 [–.07, .06]	− .12*** [–.18, –.06]	–.30*** [–.35, –.24]	.03 [–.15, .10]	–.31*** [–.37, –.25]	–.34*** [–.40, –.28]	.02 [–.05, .08]	–.19*** [–.25, –.12]	.06 [–.01, .13]	–.11** [–.17, –.04]	.02 [–.05, .08]

*N* = 837. Displayed are Pearson product–moment correlation coefficients. BCa 95% CI = bias-corrected and accelerated bootstrap 95% confidence intervals based on 1000 bootstrap samples. TriPM = Triarchic Psychopathy Measure (Patrick, 2010; Pink et al., 2022; Roy et al., 2020). The pathological narcissism total score is comprised of the averaged 52 Pathological Narcissism Inventory (Pincus et al., 2009) items. For all correlations with gender, transgender and nonbinary participants could not be included in own gender categories due to their small numbers resulting in *n* = 833. For the relationship between gender identity activism and pathological narcissistic grandiosity as well as between gender identity activism and the dimension “enjoy hurting” of the psychopathy measure, we considered Bonferroni adjusted *p* values < .025 (two-tailed) as statistically significant; for all other correlations, we applied the conventional .05 significance level

^a^Scores were calculated using unstandardized residuals

^b^For Study 2, values of two participants are missing (*n* = 835)

^c^Coding: 1 = male, 2 = female

^*^p ≤ .05. **p ≤ .01. ***p ≤ .001. The *p* values refer to two-tailed testing

### Pre-Registered Main Analyses

As expected, we found higher pathological narcissistic grandiosity to be related to more pronounced involvement in gender identity activism (*r* = .19, *p* < .001; pre-registered Hypothesis 4). Spearman’s nonparametric correlation coefficient for this association was even higher (ρ = .21, *p* < .001). We did not, however, find a significant association between higher psychopathy “enjoy hurting” and involvement in gender identity activism (*r* = –.03, *p* = .352; ρ = .05, *p* = .118; pre-registered Hypothesis 7).^3^

In a next step, we regressed involvement in gender identity activism on the two pathological narcissism facets (i.e., grandiosity and vulnerability) as well as the seven psychopathy facets (i.e., leadership, immunity to stress, positive self, callous, enjoy hurting, callous, impulsivity, antisociality). In line with our pre-registered Hypothesis 4, we found higher pathological narcissistic grandiosity to predict greater involvement in gender identity activism (β = .13, *p* = .014). Contradicting pre-registered Hypothesis 7, we found psychopathy “enjoy hurting” to be a significantly *negative* predictor of involvement in gender identity activism (β = –.11, *p* = .017). Interestingly, this analysis also revealed psychopathy “callous” to be a significant negative predictor of involvement in gender identity activism (β = –.13, *p* = .001). Also, we found a significant *positive* effect of psychopathy “antisociality” (β = .11, *p* = .005). For all results of this regression analysis, see Table 7.

**Table 7:** Multiple regression analysis for involvement in gender identity activism for Study 2

	*B*	*SE*	BCa 95% CI for *B*	β	*t*	*p*	*R*^*2*^	*df1, df2*	*F*	*p*
							.07	9, 827	7.27	< .001
Constant	1.19	0.24	0.75, 1.62	–	5.01	< .001				
Pathological narcissistic grandiosity	0.12	0.05	0.02, 0.21	.13	2.47	.014				
Pathological narcissistic vulnerability	0.03	0.05	–0.08, 0.15	.04	0.67	.506				
TriPM leader	0.04	0.05	–0.06, 0.13	.03	0.71	.479				
TriPM stress immune	–0.01	0.06	–0.12, 0.11	–.01	–0.10	.919				
TriPM positive self	0.01	0.05	–0.08, 0.09	.01	0.23	.815				
TriPM callous	–0.16	0.05	–0.26, –0.07	–.13	–3.29	.001				
TriPM enjoy hurting	–0.19	0.08	–0.33, –0.03	–.11	–2.39	.017				
TriPM impulsive	0.08	0.06	–0.04, 0.19	.06	1.34	.182				
TriPM antisocial	0.21	0.08	0.06, 0.38	.11	2.79	.005				

*N* = 837. The sample sizes are reduced as transgender and nonbinary participants could not be included in own gender categories due to their small numbers. BCa 95% CI = bias-corrected and accelerated bootstrap 95% confidence intervals based on 1000 bootstrap samples. The *p* values in the table refer to two-tailed testing. For the relationship between gender identity activism and pathological narcissistic grandiosity as well as between gender identity activism and the dimension “enjoy hurting” of the psychopathy measure, we considered Bonferroni adjusted *p* values < .025 (two-tailed) as statistically significant

### Pre-Registered Secondary Analyses

Further, we examined whether there was an indirect association between pathological narcissistic grandiosity and involvement in gender identity activism via virtue signaling (pre-registered Hypothesis 5) and aggression (pre-registered Hypothesis 6). Again, we used PROCESS (model 4—parallel multiple mediator model; Hayes, 2022). In line with our pre-registered Hypothesis 5, we found a significant effect of pathological narcissistic grandiosity on virtue signaling (*a* = 0.27, 95% BCI [0.19, 0.34]). Also, results revealed a significant direct (*c*’ = 0.15, 95% BCI [0.08, 0.22]) and indirect effect (*ab* = 0.04, 95% BCI [0.02, 0.06]) between pathological narcissistic grandiosity and involvement in gender identity activism via virtue signaling. Further, we found a significant effect of pathological narcissistic grandiosity on aggression (*a* = 0.44, 95% BCI [0.36, 0.52]). Unexpectedly, in contrast to our pre-registered Hypothesis 6 we did not find a significant indirect path between pathological narcissistic grandiosity and involvement in gender identity activism via aggression (*ab* = –0.01, 95% BCI [–0.04, 0.01]).

Next, we tested pre-registered Hypothesis 8 by looking for an indirect relationship between the psychopathy subdimension “enjoy hurting” and involvement in gender identity activism via aggression. Using PROCESS (model 4—single mediator model; Hayes, 2022), results revealed a significant path from psychopathy “enjoy hurting” to aggression (*a* = 1.02, 95% BCI [0.87, 1.16]). Nevertheless, we found neither a statistically significant direct (*c*’ = –0.10, 95% BCI [–0.21, 0.02]) nor indirect effect (*ab* = 0.04, 95% BCI [–0.01, 0.11]) between psychopathy “enjoy hurting” and involvement in gender identity activism via aggression. For all results, see Table 8.

**Table 8:** Multiple regression analysis for involvement in gender identity activism via virtue signaling and/or aggression for Study 2

Variable	Consequent														
	*M*_1_ (Aggression)					*M*_2_ (Virtue signaling)					Involvement in Gender Identity Activism				
	*B*	95% BCI for *B*	*SE B*	*t*	*p*	*B*	95% BCI for *B*	*SE B*	*t*	*p*	*B*	95% BCI for *B*	*SE B*	*t*	*p*
Constant	2.02	1.84, 2.20	0.09	21.80	< .001	–0.59	–0.77, –0.41	0.08	–7.05	< .001	1.29	1.12, 1.44	0.09	13.87	< .001
Pathological narcissistic grandiosity	0.44	0.36, 0.52	0.04	11.27	< .001	0.27	0.19, 0.34	0.04	7.53	< .001	0.15	0.08, 0.22	0.03	4.30	< .001
*M*_1_ (Aggression)	–	–	–	–	–	–	–	–	–	–	–0.03	–0.08, 0.03	0.03	–0.97	.334
*M*_2_ (Virtue signaling)	–	–	–	–	–	–	–	–	–	–	0.14	0.08, 0.21	0.03	4.76	< .001
		*R*^2^ = .13 *F*(1, 835) = 127.01, *p* < .001					*R*^2^ = .06 *F*(1, 835) = 56.73, *p* < .001					*R*^2^ = .06 *F*(3, 833) = 18.68, *p* < .001			

	*M* (Aggression)					Involvement in Gender Identity Activism				
	*B*	95% BCI for *B*	*SE B*	*t*	*p*	*B*	95% BCI for *B*	*SE B*	*t*	*p*
Constant	1.51	1.30, 1.72	0.11	14.11	< .001	1.55	1.37, 1.74	0.10	15.35	< .001
TriPM enjoyment of hurting	1.02	0.87, 1.16	0.07	14.49	< .001	–0.10	–0.21, 0.02	0.07	–1.48	.141
*M* (Aggression)		–	–	–	–	0.04	–0.01, 0.10	0.03	1.43	.153
		*R*^2^ = .20 *F*(1, 835) = 210.01, *p* < .001					*R*^2^ = .00 *F*(2, 834) = 1.46, *p* = .233			

95% BCI = bootstrap 95% confidence intervals based on 1000 bootstrap samples. For the relationship between gender identity activism and pathological narcissistic grandiosity as well as between gender identity activism and the psychopathy dimension “enjoy hurting,” we considered Bonferroni adjusted *p* values < .025 (two-tailed) as statistically significant. The *p* values in the table refer to two-tailed testing

## Discussion

The results of Study 2 demonstrated further evidence for the narcissism–activism association in the context of gender identity activism. As the sample included only a few individuals self-identifying as transgender or nonbinary, these results hold mainly for their allies. Contrary to our predictions, we found lower psychopathy “enjoy hurting” to predict involvement in gender identity activism. Lastly, we found pathological narcissistic grandiosity and involvement in gender identity activism to be indirectly related via virtue signaling. Contrary to our expectations, we did not find any evidence that aggression plays an important role in the narcissism/psychopathy–activism relationship, indicating that displays of aggression seem to be the exception in the context of gender identity activism.

## General Discussion

In the present research, we wanted to deepen our understanding of the DEVP. For this purpose, we firstly aimed at replicating the narcissism–activism relationship found in previous studies (Bertrams & Krispenz, 2024; Krispenz & Bertrams, 2024a) within the context of LGBQ and specifically gender identity activism. Secondly, we aimed at specifying which dark personality components beyond narcissism may be relevant for the DEVP.

### The Narcissism–Activism Relationship

The results of the present research show that involvement in LGBQ activism is predicted by elevated altruism, being younger, and being female. However, the main results of both studies also indicate that individuals’ high in pathological narcissistic grandiosity may be drawn to prosocial activism. In line with our pre-registered hypotheses, we found that higher pathological narcissistic grandiosity was related to higher involvement in LBGQ activism (Study 1) as well as gender identity activism (Study 2). Both times, the size of the relationships was at the level of the average effect typically found in individual differences research ($\bar{r}$ = .19; Gignac & Szodorai, 2016) and social psychological research ($\bar{r}$ = .21; Richard et al., 2003). Also, the size of these relationships was higher than the median of the effect sizes found in other pre-registered studies (*Mdn* = .16; Schäfer & Schwarz, 2019). According to Funder and Ozer (2019), “[…] an effect size *r* of .05 indicates an effect that is very small for the explanation of single events but potentially consequential in the not-very-long run, an effect size *r* of .10 indicates an effect that is still small at the level of single events but potentially more ultimately consequential, an effect size *r* of .20 indicates an effect of medium size that is of some explanatory and practical use even in the short run and therefore even more important…” (p. 166). Therefore, we consider our findings regarding the relationship between pathological narcissistic grandiosity and activism relevant. We interpret these results as further preliminary empirical evidence for the validity of the DEVP. The DEVP assumes that individuals with pronounced dark personalities may repurpose political and social activism to use it as a vehicle to satisfy their ego-focused needs rather than for the pursuit of prosocial goals and social justice. Narcissistic grandiosity is characterized by entitlement, inflated self-image, fantasies of unlimited power, superiority, perfection, and adulation as well as exploitation and lack of empathy (Wright et al., 2010). Additionally, in contrast to nonclinical conceptualizations, pathological narcissistic grandiosity is associated with higher hostility, suspiciousness, cognitive/perceptual dysregulation, emotional lability, irresponsibility, eccentricity, and rigid perfectionism (Miller et al., 2014)—attributes that may lead to problematic expressions of political or social activism and undermine prosocial activist goals. The results of the present research also replicate the findings of previous studies which found higher narcissistic traits to predict involvement in anti-sexual assault activism (Bertrams & Krispenz, 2024), feminist activism (Krispenz & Bertrams, 2024a), climate activism (Zacher, 2024), and political activism (Fazekas & Hatemi, 2021; Krispenz & Bertrams, 2024b; Rogoza et al., 2022).

We had argued that the moral-based elements of prosocial activism may provide narcissistic individuals with an opportunity for signaling their self-accredited grandiose moral superiority and virtue. As LGBQ and gender identity activism comprise moral-based elements (e.g., equality and social justice; Dunn & Szymanski, 2018), we were able to empirically test this argument. The results of the present research provide direct empirical evidence for this notion: Replicating the findings of previous research (Konrath et al., 2016; Ok et al., 2021), we did not only find a relationship between pathological narcissistic grandiosity and virtue signaling; our results also suggest that pathological narcissistic grandiosity is indirectly related to involvement in LGBQ activism (Study 1) and gender identity activism (Study 2) via virtue signaling. Hence, activism integrated morality seems to attract narcissistic virtue signalers enabling them to inauthentically signal their self-accredited prosocial virtue while truly striving for ego-focused goals, such as personal fame and social status.

In contrast, the results of the current research did not support our assumption that narcissists may use LGBQ and gender identity activism to dominate others and to engage in aggression: Even though we found pathological narcissistic grandiosity to significantly predict dominance (Study 1) as well as aggression (Study 2), we found no relationship between involvement in LGBQ/gender identity activism and dominance/aggression. These results suggest that narcissistic activists with tendencies for dominance and aggression seem to restrain from expressing those tendencies in the context of LGBQ/gender identity activism. This restriction could potentially be due the social norms prevalent in the context of LGBQ and gender identity activism. As LGBQ people are still facing discrimination and violence (e.g., James et al., 2016), the LGBQ movement advocates for openness, understanding, love, safety, and community (e.g., Ramdas, 2021). Therefore, LGBQ and gender identity activism do not seem to provide an appropriate vehicle for narcissists to express dominant or aggressive behaviors. Other forms of activism may, however, serve narcissistic activists as a better vehicle in this regard. For example, in the context of radical political activism (e.g., Costello et al., 2022; Rogoza et al., 2022), group norms seem to be in strong support of violent actions (Lenihan, 2022). Hence, future research is needed to decipher the relationship between activism, the normative endorsement of violence, and the narcissistic expression of dominant and/or aggressive tendencies.

In the current political climate, it is necessary to stress that the DEVP does *not* mean that activism is narcissistic per se. Many of those who participate in LGBQ and gender identity activism will be authentically motivated by the historic and contemporary facts about the discrimination of LGBQ and transgender people. Empirical evidence for the altruistic motives of many activists was already found previously (Bertrams & Krispenz, 2024; Krispenz & Bertrams, 2024a). The present research further supports this notion: While Study 1 showed that altruism was a predictor of involvement in LGBQ activism next to pathological narcissistic grandiosity, the results of Study 2 indirectly suggest that involvement in gender identity activism is driven by empathy and kindness (rather than the psychopathic traits of enjoying hurting others and callousness).

Most importantly, we do not assume that the DEVP holds for specific groups (e.g., members of the LGBQ community). This is supported by the results of Study 2 which included only four transgender or nonbinary individuals. This fact allowed us to investigate the narcissism–activism relationship among individuals in support of transgender or nonbinary people who do not themselves belong to the minority group (i.e., allies; Jones et al., 2014). Nevertheless, we were able to replicate the narcissism–activism relationship as predicted by the DEVP. These results further stress the vehicle aspect of the DEVP which assumes that some forms of activism may be hijacked by grandiose narcissists pretending to be allies of the minority while striving to satisfy their ego-focused needs.

### The Relationship between Activism and Other Dark Personality Traits

Further, we aimed at specifying which dark personality components may be relevant for the DEVP beyond narcissism. This side examination was primarily intended to inform future research. In Study 1, we explored the relationship between the dark personality variables of the Dark Tetrad (Paulhus et al., 2021) and LGBQ activism. The results showed that greater involvement in LGBQ activism was related to higher Dark Tetrad psychopathy and lower Dark Tetrad sadism but not to Dark Tetrad Machiavellianism. One possible explanation for these results is that involvement in LGBQ activism may provide opportunities to act psychopathically (e.g., by publicly shaming political opponents); at the same time, the role of an activist may inhibit possibilities to comfortably act sadistically (e.g., by observing such incidents from an uninvolved position). LGBQ activism does, however, not seem to provide a vehicle for Machiavellians to act in a calculating manner. In Study 2, we focused on the psychopathy–activism relationship to investigate the DEVP and assumed higher involvement in gender identity activism to be related to higher psychopathic enjoyment of hurting others. The results of Study 2 did not provide evidence for our prediction but even pointed into the opposite direction as they showed that lower psychopathy “enjoy hurting” predicts higher involvement in gender identity activism. Interestingly, this analysis also revealed psychopathy “callous” to be a significantly negative predictor of involvement in gender identity activism. These results could be due to a different conceptualization of psychopathy: While Krispenz and Bertrams (2024b) as well as Study 1 of the present research conceptualized psychopathy as part of the dark triad/tetrad, Study 2 focused on the seven-factor model of psychopathy (Pink et al., 2022; Roy et al., 2020). This model assumes that both factors—the enjoyment of hurting others and callousness—represent the meanness component of psychopathy which itself is characterized by aggressive and cruel behaviors as well as the lack of empathy and sensitivity toward others. In this light, the results of Study 2 could be mirroring the opposite of meanness (i.e., empathy and kindness) and, therefore, the prosocial aspect of involvement in gender identity activism. As these results show that individuals who are more involved in gender identity activism seem to be kinder (i.e., do not enjoy hurting others) and more empathic (i.e., less callous), they are further evidence that activism is not per se psychopathic. Another possible explanation is that gender identity activism does restrain psychopathic individuals from openly hurting others for their own enjoyment due the prevalent social norms. Nevertheless, it is worth noting that the found effects are rather small. Hence, more research is needed to decipher the psychopathy–activism relationship.

### Strengths and Limitations

The DEVP is not yet an elaborated theory. Rather, we understand the present research as part of the process of theory construction methodology (Borsboom et al., 2021)*.* This methodology has a bottom-up nature in which inquiry moves from phenomena to explanation. Within Borsboom et al.’s (2021) theoretical circle, our research on the DEVP is still at the point where we need to reliably identify the new empirical phenomenon by means of data collection. One of the strengths of the present research is the pre-registration and the publication of the complete study material and raw data. Therefore, we consider this research as a part of strong psychological science (Fiedler, 2017). Moreover, we declare that until now (2024, 1 July), we have conducted several empirical studies on the DEVP. All these studies were pre-registered, made public, and identified the expected narcissism–activism relationships. Thus, there is no file drawer with empirical studies falsifying the DEVP which makes us confident that the DEVP represents a fruitful research approach.

We nevertheless acknowledge that there still are open questions currently limiting the conceptual strength of the DEVP: For which kinds of activism is the DEVP (not) valid? Do different forms of activism provide different vehicles for narcissistic individuals due to the specific social norms prevalent in different forms of activism? Which other dark personality factors next to narcissism and psychopathy are relevant for which kind of activism, and why? Viewed from a more abstract perspective, another objective for future research on the DEVP is to determine variables moderating the narcissism–activism relationship. We also believe that the DEVP holds independently of any political orientation. To test this assumption, future research should investigate the validity of the principle including individuals on both sides of the political spectrum (for example in the context of abortion rights or gun control laws).

### Practical Implications

Our results suggest that the controversies fought out in the name of pro-LGBQ and/or pro-gender identity activism may only on the surface be related to conflicts around sexual/gender identity while they are actually caused—at least in part—by the dark traits of some activists who are hijacking the activist movement for the satisfaction of their own ego-focused needs (e.g., to signal their self-accredited moral superiority). The involvement of narcissistic or psychopathic individuals in such activism can have detrimental practical consequences (e.g., for the gender identity movement and the respective minority). For example, narcissistic individuals may strive for influential positions in the movement (e.g., Mahadevan & Jordan, 2022) and morally inflate themselves (Ok et al., 2021). Instead of raising awareness, such behavior may cause irreparable reputational harm to the activist movement inevitably reducing public support for the cause. Therefore, minorities and their true allies need to be aware of the narcissistic “enemies from within” their movement so they can take adequate measures to protect their activist movement.

## Supplementary Information

Below is the link to the electronic supplementary material.

Supplementary file1 (DOCX 85 KB)

## Data Availability

Prior to the data collection, the hypothesis and analysis plan for the current research were pre-registered at https://aspredicted.org. The pre-registrations, full datasets, and complete materials of the current studies are available at https://researchbox.org/778 (Study 1) and https://researchbox.org/970 (Study 2).

## Footnotes

1 The acronym “TERF” stands for trans-exclusionary radical feminist and has a derogatory connotation. https://en.wikipedia.org/wiki/TERF_(acronym)).

2 When sexual orientation and self-identification as a member of the LGBQ community were included as additional covariates into the analysis, similar results were obtained. Interestingly, self-identification as a member of the LGBQ community also emerged as a statistically significant predictor of involvement in LGBQ activism measured at the first time of measurement (β = .36, *p* < .001) and at the follow-up data collection (β = .34, *p* = .005).

3 The results of Study 1 showed that the narcissism–activism relationship held above and beyond the relevant covariates. Therefore, we did not pre-register such an analysis for Study 2. However, repeating this analysis by regressing involvement in gender identity activism on pathological narcissistic grandiosity and the available covariates (i.e., pathological narcissistic vulnerability, age, gender, sexual orientation) showed that the relationship between involvement in gender identity activism and pathological narcissistic grandiosity was statistically significant (β = .15, *p* < .001) above and beyond these covariates.

## References

[CR1] Anderson, C. A., & Bushman, B. J. (2002). Human aggression. *Annual Review of Psychology,**53*, 27–51. 10.1146/annurev.psych.53.100901.135231 11752478 10.1146/annurev.psych.53.100901.135231

[CR2] Aquino, K., & Reed, A., II. (2002). The self-importance of moral identity. *Journal of Personality and Social Psychology,**83*(6), 1423–1440. 10.1037/0022-3514.83.6.1423 12500822 10.1037//0022-3514.83.6.1423

[CR3] Atteberry Ash, B. (2020). *Social work, social justice, and the causes to which we are called: Attitudes, ally behavior, and activism*. Electronic Theses and Dissertations. https://digitalcommons.du.edu/etd/1718

[CR48] BBC News. (2021, July 5). *Georgia: Tbilisi Pride cancelled amid violent protests.* https://www.bbc.com/news/world-europe-57720366

[CR4] Bertrams, A., & Krispenz, A. (2024). Dark-ego-vehicle principle: Narcissism as a predictor of anti-sexual assault activism. *Current Psychology,**43*, 3585–3589. 10.1007/s12144-023-04591-4

[CR5] Bjork-James, S. (2019). Christian nationalism and LGBTQ structural violence in the United States. *Journal of Religion and Violence,**7*(3), 278–302. 10.5840/jrv202031069

[CR6] Borsboom, D., van der Maas, H. L. J., Dalege, J., Kievit, R. A., & Haig, B. D. (2021). Theory construction methodology: A practical framework for building theories in psychology. *Perspectives on Psychological Science,**16*(4), 756–766. 10.1177/1745691620969647 33593167 10.1177/1745691620969647

[CR7] Bratton, T. M., Lyle, R., & ten Besel, T. (2020). Attitudes of Muslim Americans toward homosexuality and marriage equality: Moving beyond simply understanding Christian public opinions. *Sociological Inquiry,**90*(4), 765–793. 10.1111/soin.12334

[CR8] Bushman, B., & Thomaes, S. (2011). When the narcissistic ego deflates, narcissistic aggression inflates. In W. K. Campbell & J. D. Miller (Eds.), *The handbook of narcissism and narcissistic personality disorder: Theoretical approaches, empirical findings, and treatments* (pp. 319–329). Wiley. 10.1002/9781118093108.ch28

[CR9] Campbell, W. K., Bush, C. P., Brunell, A. B., & Shelton, J. (2005). Understanding the social costs of narcissism: The case of the tragedy of the commons. *Personality and Social Psychology Bulletin,**31*(10), 1358–1368. 10.1177/0146167205274855 16143668 10.1177/0146167205274855

[CR10] Caruso, S. (2023, June 30). Everything to know about the Bud Light controversy. *People*. https://people.com/bud-light-controversy-everything-to-know-7547159

[CR11] Chang, S.-J., van Witteloostuijn, A., & Eden, L. (2010). From the editors: Common method variance in international business research. *Journal of International Business Studies,**41*(2), 178–184. 10.1057/jibs.2009.88

[CR12] Cheng, J. T., Tracy, J. L., & Henrich, J. (2010). Pride, personality, and the evolutionary foundations of human social status. *Evolution and Human Behavior,**31*(5), 334–347. 10.1016/j.evolhumbehav.2010.02.004

[CR13] Costello, T. H., Bowes, S. M., Stevens, S. T., Waldman, I. W., Tasimi, A., & Lilienfeld, S. O. (2022). Clarifying the structure and nature of left-wing authoritarianism. *Journal of Personality and Social Psychology,**122*(1), 135–170. 10.1037/pspp0000341 34383522 10.1037/pspp0000341

[CR14] Crowe, M. L., & Miller, J. D. (2023). Trait and state measurement of grandiose and vulnerable narcissism. In P. K. Jonason (Ed.), *Shining light on the dark side of personality* (pp. 32–43). Hogrefe.

[CR15] Dargan, S., & Schermer, J. A. (2022). Predicting altruism with personality “beyond” the Big Five. *Personality and Individual Differences,**185*, 111258. 10.1016/j.paid.2021.111258

[CR16] Donnellan, M. B., & Burt, S. A. (2016). A further evaluation of the triarchic conceptualization of psychopathy in college students. *Journal of Psychopathology and Behavioral Assessment,**38*, 172–182. 10.1007/s10862-015-9512-z

[CR17] Dunn, T. L., & Szymanski, D. M. (2018). Heterosexist discrimination and LGBQ activism: Examining a moderated mediation model. *Psychology of Sexual Orientation and Gender Diversity,**5*(1), 13–24. 10.1037/sgd0000250

[CR18] Faul, F., Erdfelder, E., Buchner, A., & Lang, A.-G. (2009). Statistical power analyses using G*Power 3.1: Tests for correlation and regression analyses. *Behavior Research Methods,**41*, 1149–1160. 10.3758/BRM.41.4.1149 19897823 10.3758/BRM.41.4.1149

[CR19] Fazekas, Z., & Hatemi, P. K. (2021). Narcissism in political participation. *Personality and Social Psychology Bulletin,**47*(3), 347–361. 10.1177/0146167220919212 32493116 10.1177/0146167220919212

[CR20] Fiedler, K. (2017). What constitutes strong psychological science? The (neglected) role of diagnosticity and a priori theorizing. *Perspectives on Psychological Science,**12*(1), 46–61. 10.1177/1745691616654458 28073328 10.1177/1745691616654458

[CR21] Field, A. (2018). *Discovering statistics using IBM SPSS Statistics* (5th ed.). Sage

[CR23] Funder, D. C., & Ozer, D. J. (2019). Evaluating effect size in psychological research: Sense and nonsense. *Advances in Methods and Practices in Psychological Science,**2*(2), 156–168. 10.1177/2515245919847202

[CR24] Furnham, A., Richards, S. C., & Paulhus, D. L. (2013). The dark triad of personality: A 10 year review. *Social and Personality Psychology Compass,**7*(3), 199–216. 10.1111/spc3.12018

[CR25] Gignac, G. E., & Szodorai, E. T. (2016). Effect size guidelines for individual differences researchers. *Personality and Individual Differences,**102*, 74–78. 10.1016/j.paid.2016.06.069

[CR26] Gray, N. S., Blumenthal, S., Shuker, R., Wood, H., Fonagy, P., & Snowden, R. J. (2019). The triarchic model of psychopathy and antisocial behavior: Results from an offender population with personality disorder. *Journal of Interpersonal Violence,**36*(17–18), NP9130–NP9152. 10.1177/0886260519853404 31189393 10.1177/0886260519853404

[CR27] Greenwood, D., Long, C. R., & Dal Cin, S. (2013). Fame and the social self: The need to belong, narcissism, and relatedness predict the appeal of fame. *Personality and Individual Differences,**55*(5), 490–495. 10.1016/j.paid.2013.04.020

[CR28] Grijalva, E., Harms, P. D., Newman, D. A., Gaddis, B. H., & Fraley, R. C. (2015). Narcissism and leadership: A meta-analytic review of linear and nonlinear relationships. *Personnel Psychology,**68*(1), 1–47. 10.1111/peps.12072

[CR29] Hayes, A. F. (2022). *Introduction to mediation, moderation, and conditional process analysis: A regression-based approach* (3rd ed.). Guilford Press

[CR30] Hepper, E. G., Hart, C. M., Meek, R., Cisek, S., & Sedikides, C. (2014). Narcissism and empathy in young offenders and non-offenders. *European Journal of Personality,**28*(2), 201–210. 10.1002/per.1939

[CR31] Hruska, J., & Maresova, P. (2020). Use of social media platforms among adults in the United States—behavior on social media. *Societies,**10*, 27. 10.3390/soc10010027

[CR32] James, S. E., Herman, J. L., Rankin, S., Keisling, M., Mottet, L., & Anafi, M. (2016). *The report of the 2015 U.S. Transgender Survey*. Washington, DC: National Center for Transgender Equality. https://transequality.org/sites/default/files/docs/usts/USTS%20Full%20Report%20-%20FINAL%201.6.17.pdf

[CR33] Jones, D. N., & Paulhus, D. L. (2014). Introducing the short dark triad (SD3): A brief measure of dark personality traits. *Assessment,**21*(1), 28–41. 10.1177/1073191113514105 24322012 10.1177/1073191113514105

[CR34] Jones, K. N., Brewster, M. E., & Jones, J. A. (2014). The creation and validation of the LGBT Ally Identity Measure. *Psychology of Sexual Orientation and Gender Diversity,**1*(2), 181–195. 10.1037/sgd0000033

[CR35] Kelleher, P. (2021, October 7). Students demand Sussex uni fire anti-trans professor Kathleen Stock: “We’ve f**cking had enough”. *Pink News*. https://www.thepinknews.com/2021/10/07/kathleen-stock-university-sussex/

[CR36] Kjærvik, S. L., & Bushman, B. J. (2021). The link between narcissism and aggression: A meta-analytic review. *Psychological Bulletin,**147*(5), 477–503. 10.1037/bul0000323 34292012 10.1037/bul0000323

[CR37] Konrath, S., Corneille, O., Bushman, B. J., & Luminet, O. (2014). The relationship between narcissistic exploitativeness, dispositional empathy, and emotion recognition abilities. *Journal of Nonverbal Behavior,**38*(1), 129–143. 10.1007/s10919-013-0164-y

[CR38] Konrath, S., Ho, M.-H., & Zarins, S. (2016). The strategic helper: Narcissism and prosocial motives and behaviors. *Current Psychology,**35*(2), 182–194. 10.1007/s12144-016-9417-3

[CR39] Krispenz, A., & Bertrams, A. (2024a). Further basic evidence for the dark-ego-vehicle principle: Higher pathological narcissism is associated with greater involvement in feminist activism. *Current Psychology,**43*, 14619–14633. 10.1007/s12144-023-05451-x

[CR40] Krispenz, A., & Bertrams, A. (2024b). Understanding left-wing authoritarianism: Relations to the dark personality traits, altruism, and social justice commitment. *Current Psychology,**43*, 2714–2730. 10.1007/s12144-023-04463-x

[CR41] Krizan, Z., & Herlache, A. D. (2018). The narcissism spectrum model: A synthetic view of narcissistic personality. *Personality and Social Psychology Review,**22*(1), 3–31. 10.1177/1088868316685018 28132598 10.1177/1088868316685018

[CR42] Lenihan, E. (2022). A classification of Antifa Twitter accounts based on social network mapping and linguistic analysis. *Social Network Analysis and Mining,**12*. 10.1007/s13278-021-00847-8

[CR43] Lindell, M. K., & Whitney, D. J. (2001). Accounting for common method variance in cross-sectional research designs. *Journal of Applied Psychology,**86*(1), 114–121. 10.1037/0021-9010.86.1.114 11302223 10.1037/0021-9010.86.1.114

[CR44] Mahadevan, N., & Jordan, C. (2022). Desperately seeking status: How desires for, and perceived attainment of, status and inclusion relate to grandiose and vulnerable narcissism. *Personality and Social Psychology Bulletin,**48*(5), 704–717. 10.1177/01461672211021189 34112051 10.1177/01461672211021189 PMC9066682

[CR45] Miller, J. D., McCain, J., Lynam, D. R., Few, L. R., Gentile, B., MacKillop, J., & Campbell, W. K. (2014). A comparison of the criterion validity of popular measures of narcissism and narcissistic personality disorder via the use of expert ratings. *Psychological Assessment,**26*(3), 958–969. 10.1037/a0036613 24773036 10.1037/a0036613

[CR46] Neumann, C. S., Hare, R. D., & Pardini, D. A. (2015). Antisociality and the construct of psychopathy: Data from across the globe. *Journal of Personality,**83*, 678–692. 10.1111/jopy.12127 25181550 10.1111/jopy.12127

[CR47] Nevicka, B. (2018). Narcissism and leadership: A perfect match? In A. Hermann, A. Brunell, & J. Foster (Eds.), *Handbook of trait narcissism* (pp. 399–407). Springer. 10.1007/978-3-319-92171-6_43

[CR49] Ok, E., Qian, Y., Strejcek, B., & Aquino, K. (2021). Signaling virtuous victimhood as indicators of Dark Triad personalities. *Journal of Personality and Social Psychology,**120*(6), 1634–1661. 10.1037/pspp0000329 32614222 10.1037/pspp0000329

[CR50] Patrick, C. J. (2010). *Operationalizing the triarchic conceptualization of psychopathy: Preliminary description of brief scales for assessment of boldness, meanness, and disinhibition.* Unpublished manual, Department of Psychology, Florida State University

[CR51] Paulhus, D. L., Buckels, E. E., Trapnell, P. D., & Jones, D. N. (2021). Screening for dark personalities: The Short Dark Tetrad (SD4). *European Journal of Psychological Assessment,**37*(3), 208–222. 10.1027/1015-5759/a000602

[CR52] Pincus, A. L. (2023). A brief overview of the Pathological Narcissism Inventory. In P. K. Jonason (Ed.), *Shining light on the dark side of personality* (pp. 9–21). Hogrefe.

[CR53] Pincus, A. L., Ansell, E. B., Pimentel, C. A., Cain, N. M., Wright, A. G. C., & Levy, K. N. (2009). Initial construction and validation of the Pathological Narcissism Inventory. *Psychological Assessment,**21*(3), 365–379. 10.1037/a0016530 19719348 10.1037/a0016530

[CR54] Pink, J., Snowden, R. J., Price, M. J., Kocsondi, A., Lawrence, C., Stephens, P., White, W., & Gray, N. S. (2022). Refining the relationship between psychopathy, aggression, and rule-breaking by gender: A comparison of the triarchic and septarchic models of psychopathy. *Personality and Individual Differences,**185*, 111282. 10.1016/j.paid.2021.111282

[CR55] Ramdas, K. (2021). Negotiating LGBTQ rights in Singapore: The margin as a place of refusal. *Urban Studies,**58*(7), 1448–1462. 10.1177/0042098020962936

[CR56] Reid, G. (2023, May 17). *Russia, homophobia and the battle for “traditional values”*. Human Rights Watch. https://www.hrw.org/news/2023/05/17/russia-homophobia-and-battle-traditional-values

[CR57] Reynolds, J. (2023, August 13). Sarah Jane Baker: Trans activist cleared of inciting violence. *BBC*. https://www.bbc.com/news/uk-england-london-66676737

[CR58] Richard, F. D., Bond, C. F., Jr., & Stokes-Zoota, J. J. (2003). One hundred years of social psychology quantitatively described. *Review of General Psychology,**7*(4), 331–363. 10.1037/1089-2680.7.4.331

[CR59] Rogoza, M., Marchlewska, M., & Szczepańska, D. (2022). Why dark personalities participate in politics? *Personality and Individual Differences,**186*, 111319. 10.1016/j.paid.2021.111319

[CR60] Roy, S., Vize, C., Uzieblo, K., van Dongen, J. D. M., Miller, J., Lynam, D., Brazil, I., Yoon, D., Mokros, A., Gray, N. S., Snowden, R., & Neumann, C. S. (2020). Triarchic or septarchic?—uncovering the triarchic psychopathy measure’s (TriPM) structure. *Personality Disorders, Theory, Research, and Treatment,**12*(1), 1–15. 10.1037/per0000392 10.1037/per0000392 31971417

[CR61] Rushton, J. P., Chrisjohn, R. D., & Fekken, G. C. (1981). The altruistic personality and the Self-Report Altruism Scale. *Personality and Individual Differences,**2*(4), 293–302. 10.1016/0191-8869(81)90084-2

[CR62] Russell, E. L. (2019). *The discursive ecology of homophobia: Unraveling anti-LGBTQ speech on the European far right* (Vol. 16). Multilingual Matters

[CR63] Schäfer, T., & Schwarz, M. A. (2019). The meaningfulness of effect sizes in psychological research: Differences between sub-disciplines and the impact of potential biases. *Frontiers in Psychology,**10*, 813. 10.3389/fpsyg.2019.00813 31031679 10.3389/fpsyg.2019.00813 PMC6470248

[CR64] Schönbrodt, F. D., & Perugini, M. (2013). At what sample size do correlations stabilize? *Journal of Research in Personality,**47*, 609–612. 10.1016/j.jrp.2013.05.009

[CR65] Strauss Swanson, C., & Szymanski, D. M. (2021). Anti-sexual assault activism and positive psychological functioning among survivors. *Sex Roles,**85*, 25–38. 10.1007/s11199-020-01202-5

[CR66] Szymanski, D. M., Goates, J. D., & Strauss Swanson, C. (2021). LGBQ activism and positive psychological functioning: The roles of meaning, community connection, and coping. *Psychology of Sexual Orientation and Gender Diversity,**10*(1), 70–79. 10.1037/sgd0000499

[CR67] Tabachnick, B. G., & Fidell, L. S. (2007). *Using multivariate statistics* (5th ed.). Allyn and Bacon

[CR68] Todd, J. (2023, April 24). *HRC mourns Koko Da Doll, Kokomo City” documentary start, killed by gun violence in Atlanta*. Human Rights Campaign. https://www.hrc.org/news/hrc-mourns-koko-da-doll-kokomo-city-documentary-star-killed-by-gun-violence-in-atlanta

[CR69] Wagner, W., & Hayes, N. (2022). Repressive moralism: World making and petty fascism in transgender politics. *Integrative Psychological and Behavioral Science,**56*(3), 573–589. 10.1007/s12124-021-09670-4 34988872 10.1007/s12124-021-09670-4

[CR70] Webster, G. D., DeWall, C. N., Pond, R. S., Jr., Deckman, T., Jonason, P. K., Le, B. M., Nichols, A. L., Orozco Schember, T., Crysel, L. C., Crosier, B. S., Smith, C. V., Paddock, E. L., Nezlek, J. B., Kirkpatrick, L. A., Bryan, A. D., & Bator, R. J. (2015). The Brief Aggression Questionnaire: Structure, validity, reliability, and generalizability. *Journal of Personality Assessment,**97*(6), 638–649. 10.1080/00223891.2015.1044093 26055531 10.1080/00223891.2015.1044093

[CR71] Wood, S., Michaelides, G., Daniels, K., & Niven, K. (2022). Uncertainty and well-being amongst homeworkers in the COVID-19 pandemic: A longitudinal study of university staff. *International Journal of Environmental Research and Public Health,**19*, 10435. 10.3390/ijerph191610435 36012069 10.3390/ijerph191610435 PMC9408406

[CR72] Woolcock, N. (2021, October 7). Sussex university students campaign to have “transphobic” professor Kathleen Stock sacked. *The Times*. https://archive.ph/20211007214040/https://www.thetimes.co.uk/article/sussex-university-students-campaign-to-have-transphobic-professor-kathleen-stock-sacked-qpn82tpbh

[CR73] Wright, A. G. C., Lukowitsky, M. R., Pincus, A. L., & Conroy, D. E. (2010). The higher order factor structure and gender invariance of the Pathological Narcissism Inventory. *Assessment,**17*(4), 467–483. 10.1177/1073191110373227 20634422 10.1177/1073191110373227

[CR74] Zacher, H. (2024). The dark side of environmental activism. *Personality and Individual Differences,**219*(1), 112506. 10.1016/j.paid.2023.112506

[CR75] Zeigler-Hill, V., Sauls, D., & Malay, P. (2021). Through the eyes of Narcissus: Competitive social worldviews mediate the associations that narcissism has with ideological attitudes. *Self and Identity,**20*(6), 811–840. 10.1080/15298868.2020.1779118

[CR76] Zhang, Y., Hou, Z., Wu, S., Li, X., Hao, M., & Wu, X. (2022). The relationship between internet addiction and aggressive behavior among adolescents during the COVID-19 pandemic: Anxiety as a mediator. *Acta Psychologica,**227*, 103612. 10.1016/j.actpsy.2022.103612 35598380 10.1016/j.actpsy.2022.103612 PMC9091340

