# Supplementary material for: Further Evidence for the Dark-Ego-Vehicle Principle: Higher Pathological Narcissistic Grandiosity and Virtue Signaling Are Related to Greater Involvement in LGBQ and Gender Identity Activism
Source: Arch Sex Behav. 2024 Dec 2;54(3):1271–91. doi: 10.1007/s10508-024-03019-9 (PMC11926010; doi:10.1007/s10508-024-03019-9)
Supplement: Supplementary file 1 — Supplementary file1 (DOCX 85 KB) [file 10508_2024_3019_MOESM1_ESM.docx]

**Further Evidence for the Dark-Ego-Vehicle Principle:**

**Higher Pathological Narcissistic Grandiosity and Virtue Signaling**

**are Related to Greater Involvement in LGBQ and Gender Identity Activism**

**Supplementary Information**

**Supplementary Table S1**

*Full Sample Demographics for Study 1 (US Sample) and Study 2 (UK Sample)*

|  | | Study 1 | | | | | | | | Study 2 | | | |
| --- | --- | --- | --- | --- | --- | --- | --- | --- | --- | --- | --- | --- | --- |
|  | | Measurement time 1 *N* = 446 | | | | Measurement time 2 *N* = 326 | | | | *N* = 837 | | | |
|  | | *n* | *M* | *SD* | % | *n* | *M* | *SD* | % | *n* | *M* | *SD* | % |
| Age (years)^a^ | |  | 46.20 | 15.98 |  |  | 48.99 | 15.34 |  |  | 47.46 | 15.20 |  |
| Gender | |  |  |  |  |  |  |  |  |  |  |  |  |
|  | Female | 225 |  |  | 50.4 | 157 |  |  | 48.2 | 438 |  |  | 52.3 |
|  | Male | 215 |  |  | 48.2 | 165 |  |  | 50.6 | 394 |  |  | 47.1 |
|  | Transgender | 3 |  |  | 0.7 | 2 |  |  | 0.6 | 1 |  |  | 0.1 |
|  | Non-binary, genderqueer, genderfluid | ­– |  |  | – | – |  |  | – | 3 |  |  | 0.4 |
|  | Other | 3 |  |  | 0.7 | 2 |  |  | 0.6 | 1 |  |  | 0.1 |
| Sexual orientation | |  |  |  |  |  |  |  |  |  |  |  |  |
|  | Heterosexual, straight | 379 |  |  | 85.0 | 286 |  |  | 87.7 | 762 |  |  | 91.0 |
|  | Homosexual, gay/lesbian | 20 |  |  | 4.5 | 15 |  |  | 4.6 | 26 |  |  | 3.1 |
|  | Bisexual | 35 |  |  | 7.8 | 20 |  |  | 6.1 | 43 |  |  | 5.1 |
|  | Other | 12 |  |  | 2.7 | 5 |  |  | 1.5 | 6 |  |  | 0.7 |
| Self-identification as a LGBQ member | |  |  |  |  |  |  |  |  |  |  |  |  |
|  | Yes | 60 |  |  | 13.5 | 38 |  |  | 11.7 | ­– |  |  | – |
|  | No | 173 |  |  | 83.6 | 283 |  |  | 86.8 | ­– |  |  | – |
|  | Unsure | 13 |  |  | 2.9 | 5 |  |  | 1.5 | ­– |  |  | – |

**Supplementary Table S1** [*continued*]

|  | | Study 1 | | | | | | | | | | | | Study 2 | | | | | |
| --- | --- | --- | --- | --- | --- | --- | --- | --- | --- | --- | --- | --- | --- | --- | --- | --- | --- | --- | --- |
|  | | Measurement time 1 *N* = 446 | | | | | Measurement time 2 *N* = 326 | | | | | | | *N* = 837 | | | | | |
|  | | *n* | | *M* | *SD* | % | *n* | *M* | *SD* | | | % | | *n* | *M* | | *SD* | | % |
| Marital status | | |  |  |  |  |  |  | |  | | |  |  | |  | |  |  |
|  | Single (never married) | | 139 |  |  | 31.2 | 101 |  | |  | | | 31.0 | 238 | |  | |  | 28.4 |
|  | Married (first marriage) | | 144 |  |  | 32.3 | 109 |  | |  | | | 33.4 | 336 | |  | |  | 40.1 |
|  | Remarried | | 32 |  |  | 7.2 | 26 |  | |  | | | 8.0 | 54 | |  | |  | 6.5 |
|  | Separated | | 13 |  |  | 2.9 | 11 |  | |  | | | 3.4 | 16 | |  | |  | 1.9 |
|  | Divorced | | 59 |  |  | 13.2 | 42 |  | |  | | | 12.9 | 52 | |  | |  | 6.2 |
|  | Widowed | | 15 |  |  | 3.7 | 14 |  | |  | | | 4.3 | 18 | |  | |  | 2.2 |
|  | Long-term domestic partner (at least one year) | | 44 |  |  | 9.9 | 23 |  | |  | | | 7.1 | 123 | |  | |  | 14.7 |
| Political orientation on left–right spectrum^b^ | |  | | 3.35 | 1.72 |  |  | 3.45 | | | 1.76 |  | |  | 3.54 | | 1.24 | |  |
| Political ideology | | |  |  |  |  |  |  | |  | | |  |  | |  | |  |  |
|  | Leftist | | 47 |  |  | 10.5 | 29 |  | |  | | | 8.9 | 122 | |  | |  | 14.6 |
|  | Liberal | | 174 |  |  | 39.0 | 126 |  | |  | | | 38.7 | 257 | |  | |  | 30.7 |
|  | Moderate | | 107 |  |  | 24.0 | 74 |  | |  | | | 22.7 | 258 | |  | |  | 30.8 |
|  | Conservative | | 103 |  |  | 23.1 | 88 |  | |  | | | 27.0 | 178 | |  | |  | 21.3 |
|  | Libertarian | | 13 |  |  | 2.9 | 8 |  | |  | | | 2.5 | 18 | |  | |  | 2.2 |
|  | Populist | | 2 |  |  | 0.4 | 1 |  | |  | | | 0.3 | 4 | |  | |  | 0.5 |
| Political party identification (US) | | |  |  |  |  |  |  | |  | | |  |  | |  | |  |  |
|  | Democratic | | 198 |  |  | 44.4 | 146 |  | |  | | | 44.8 | ­– | |  | |  | – |
|  | Green | | 4 |  |  | 0.9 | 3 |  | |  | | | 0.9 | ­– | |  | |  | – |
|  | Independent | | 99 |  |  | 22.2 | 70 |  | |  | | | 21.5 | ­– | |  | |  | – |
|  | Libertarian | | 11 |  |  | 2.5 | 7 |  | |  | | | 2.1 | ­– | |  | |  | – |
|  | Republican | | 90 |  |  | 20.2 | 76 |  | |  | | | 23.3 | ­– | |  | |  | – |
|  | Socialist | | 15 |  |  | 3.4 | 8 |  | |  | | | 2.5 | ­– | |  | |  | – |
|  | None | | 24 |  |  | 5.4 | 12 |  | |  | | | 3.7 | ­– | |  | |  | – |
|  | Other | | 5 |  |  | 1.1 | 4 |  | |  | | | 1.2 | ­– | |  | |  | – |

**Supplementary Table S1** [*continued*]

|  | | Study 1 | | | | | | | | | | | | | | | | Study 2 | | | | | | |
| --- | --- | --- | --- | --- | --- | --- | --- | --- | --- | --- | --- | --- | --- | --- | --- | --- | --- | --- | --- | --- | --- | --- | --- | --- |
|  | | Measurement time 1 *N* = 446 | | | | | | | | Measurement time 2 *N* = 326 | | | | | | | | *N* = 837 | | | | | | |
|  | | *n* | | *M* | | *SD* | | % | | *n* | | *M* | | *SD* | | % | | *n* | | *M* | | *SD* | | % |
| Political party identification (UK) | | |  | |  | |  | |  | |  | |  | |  | |  | |  | |  | |  |  |
|  | Conservative Party (Tories) | | ­– | |  | |  | | – | | ­– | |  | |  | | – | | 174 | |  | |  | 20.8 |
|  | Labour Party | | ­– | |  | |  | | – | | ­– | |  | |  | | – | | 290 | |  | |  | 34.6 |
|  | Liberal Democrats | | ­– | |  | |  | | – | | ­– | |  | |  | | – | | 90 | |  | |  | 10.8 |
|  | None | | ­– | |  | |  | | – | | ­– | |  | |  | | – | | 206 | |  | |  | 24.6 |
|  | Other | | ­– | |  | |  | | – | | ­– | |  | |  | | – | | 77 | |  | |  | 9.2 |
| Ethnicity^c^ (US) | | |  | |  | |  | |  | |  | |  | |  | |  | |  | |  | |  |  |
|  | American Indian or Alaska Native | | 3 | |  | |  | | 0.7 | | 2 | |  | |  | | 0.6 | | ­– | |  | |  | – |
|  | Asian or Asian-American | | 30 | |  | |  | | 6.7 | | 21 | |  | |  | | 6.4 | | ­– | |  | |  | – |
|  | Black or African-American | | 57 | |  | |  | | 12.8 | | 39 | |  | |  | | 12.0 | | ­– | |  | |  | – |
|  | Hispanic or Latino | | 18 | |  | |  | | 4.0 | | 11 | |  | |  | | 3.4 | | ­– | |  | |  | – |
|  | Native Hawaiian or Pacific Islander | | 1 | |  | |  | | 0.2 | | 0 | |  | |  | | 0 | | ­– | |  | |  | – |
|  | White | | 349 | |  | |  | | 78.3 | | 260 | |  | |  | | 79.8 | | ­– | |  | |  | – |
|  | Other | | 6 | |  | |  | | 1.3 | | 4 | |  | |  | | 1.2 | | ­– | |  | |  | – |
| Ethnicity^c^ (UK) | | |  | |  | |  | |  | |  | |  | |  | |  | |  | |  | |  |  |
|  | Asian or Asian-British | | ­– | |  | |  | | – | | ­– | |  | |  | | – | | 63 | |  | |  | 7.5 |
|  | Black, Black British, Caribbean, or African | | ­– | |  | |  | | – | | ­– | |  | |  | | – | | 24 | |  | |  | 2.9 |
|  | White | | ­– | |  | |  | | – | | ­– | |  | |  | | – | | 739 | |  | |  | 88.3 |
|  | Other | | ­– | |  | |  | | – | | ­– | |  | |  | | – | | 14 | |  | |  | 1.7 |
| Native language | | |  | | ­ | |  | |  | |  | |  | |  | |  | |  | |  | |  |  |
|  | English | | 416 | |  | |  | | 93.3 | | 309 | |  | |  | | 94.8 | | 767 | |  | |  | 91.6 |
|  | English and another language | | 17 | |  | |  | | 3.8 | | 12 | |  | |  | | 3.7 | | 37 | |  | |  | 4.4 |
|  | Another language | | 13 | |  | |  | | 2.9 | | 5 | |  | |  | | 1.5 | | 33 | |  | |  | 3.9 |

**Supplementary Table S1** [*continued*]

|  | | Study 1 | | | | | | | | | | | | | | | | Study 2 | | | | | | |
| --- | --- | --- | --- | --- | --- | --- | --- | --- | --- | --- | --- | --- | --- | --- | --- | --- | --- | --- | --- | --- | --- | --- | --- | --- |
|  | | Measurement time 1 *N* = 446 | | | | | | | | Measurement time 2 *N* = 326 | | | | | | | | *N* = 837 | | | | | | |
|  | | *n* | | *M* | | *SD* | | % | | *n* | | *M* | | *SD* | | % | | *n* | | *M* | | *SD* | | % |
| Highest educational level (US) | | |  | |  | |  | |  | |  | |  | |  | |  | |  | |  | |  |  |
|  | Professional degree (JD, MD) | | 12 | |  | |  | | 2.7 | | 8 | |  | |  | | 2.5 | | ­– | |  | |  | – |
|  | Doctoral degree | | 10 | |  | |  | | 2.2 | | 7 | |  | |  | | 2.1 | | ­– | |  | |  | – |
|  | Master’s degree | | 62 | |  | |  | | 13.9 | | 48 | |  | |  | | 14.7 | | ­– | |  | |  | – |
|  | Bachelor’s degree in college (four-year) | | 167 | |  | |  | | 37.4 | | 129 | |  | |  | | 39.6 | | ­– | |  | |  | – |
|  | Associate degree in college (two-year) | | 46 | |  | |  | | 10.3 | | 30 | |  | |  | | 9.2 | | ­– | |  | |  | – |
|  | Some college but no degree | | 92 | |  | |  | | 20.6 | | 61 | |  | |  | | 18.7 | | ­– | |  | |  | – |
|  | High school graduate | | 50 | |  | |  | | 11.2 | | 40 | |  | |  | | 12.3 | | ­– | |  | |  | – |
|  | Less than high school degree | | 5 | |  | |  | | 1.1 | | 2 | |  | |  | | 0.6 | | ­– | |  | |  | – |
|  | Other | | 2 | |  | |  | | 0.4 | | 1 | |  | |  | | 0.3 | | ­– | |  | |  | – |
| Highest educational level (UK) | | |  | |  | |  | |  | |  | |  | |  | |  | |  | |  | |  |  |
|  | Doctoral degree | | ­– | |  | |  | | – | | ­– | |  | |  | | – | | 20 | |  | |  | 2.4 |
|  | Master’s degree | | ­– | |  | |  | | – | | ­– | |  | |  | | – | | 131 | |  | |  | 15.7 |
|  | Bachelor’s degree | | ­– | |  | |  | | – | | ­– | |  | |  | | – | | 326 | |  | |  | 38.9 |
|  | A-levels or any equivalent | | ­– | |  | |  | | – | | ­– | |  | |  | | – | | 211 | |  | |  | 25.2 |
|  | General certificate of education or any equivalent | | ­– | |  | |  | | – | | ­– | |  | |  | | – | | 131 | |  | |  | 15.7 |
|  | Did not finish secondary education | | ­– | |  | |  | | – | | ­– | |  | |  | | – | | 3 | |  | |  | 0.4 |
|  | Other | | ­– | |  | |  | | – | | ­– | |  | |  | | – | | 15 | |  | |  | 1.8 |

**Supplementary Table S1** [*continued*]

|  | | Study 1 | | | | | | | | | | | | | | | | Study 2 | | | | | | |
| --- | --- | --- | --- | --- | --- | --- | --- | --- | --- | --- | --- | --- | --- | --- | --- | --- | --- | --- | --- | --- | --- | --- | --- | --- |
|  | | Measurement time 1 *N* = 446 | | | | | | | | Measurement time 2 *N* = 326 | | | | | | | | *N* = 837 | | | | | | |
|  | | *n* | | *M* | | *SD* | | % | | *n* | | *M* | | *SD* | | % | | *n* | | *M* | | *SD* | | % |
| Household income^d^ | | |  | |  | |  | |  | |  | |  | |  | |  | |  | |  | |  |  |
|  | Less than 10000 | | 20 | |  | |  | | 4.5 | | 13 | |  | |  | | 4.0 | | 66 | |  | |  | 7.9 |
|  | 10000 to 19999 | | 34 | |  | |  | | 7.6 | | 27 | |  | |  | | 8.3 | | 116 | |  | |  | 13.9 |
|  | 20000 to 29999 | | 52 | |  | |  | | 11.7 | | 41 | |  | |  | | 12.6 | | 163 | |  | |  | 19.5 |
|  | 30000 to 39999 | | 48 | |  | |  | | 10.8 | | 32 | |  | |  | | 9.8 | | 120 | |  | |  | 14.3 |
|  | 40000 to 49999 | | 35 | |  | |  | | 7.8 | | 28 | |  | |  | | 8.6 | | 108 | |  | |  | 12.9 |
|  | 50000 to 59999 | | 47 | |  | |  | | 10.5 | | 31 | |  | |  | | 9.5 | | 77 | |  | |  | 9.2 |
|  | 60000 to 69999 | | 30 | |  | |  | | 6.7 | | 21 | |  | |  | | 6.4 | | 61 | |  | |  | 7.3 |
|  | 70000 to 79999 | | 30 | |  | |  | | 6.7 | | 23 | |  | |  | | 7.1 | | 43 | |  | |  | 5.1 |
|  | 80000 to 89999 | | 24 | |  | |  | | 5.4 | | 19 | |  | |  | | 5.8 | | 28 | |  | |  | 3.3 |
|  | 90000 to 99999 | | 24 | |  | |  | | 5.4 | | 16 | |  | |  | | 4.9 | | 22 | |  | |  | 2.6 |
|  | 100000 to 149999 | | 68 | |  | |  | | 15.2 | | 48 | |  | |  | | 14.7 | | 25 | |  | |  | 3.0 |
|  | 150000 or more | | 34 | |  | |  | | 7.6 | | 27 | |  | |  | | 8.3 | | 8 | |  | |  | 1.0 |

*Note*. ^a^For Study 2, values of two participants are missing (*n* = 835).
^b^Selection on a seven-point scale from *extremely left-wing* (1) to *extremely right-wing* (7).

^c^The participants could select multiple choices.

^d^Household income includes the entire household income (in previous year) before taxes. US sample: values in US Dollar. UK sample: values in British Pounds Sterling.
